# Supplementary material for: Loci-specific phase separation of FET fusion oncoproteins promotes gene transcription
Source: Nat Commun. 2021 Mar 5;12:1491. doi: 10.1038/s41467-021-21690-7 (PMC7935978; doi:10.1038/s41467-021-21690-7)
Supplement: Supplementary file 1 — Supplementary Information [file 41467_2021_21690_MOESM1_ESM.pdf]

Supplementary Information

Loci-specific phase separation of FET fusion oncoproteins promotes gene transcription

Linyu Zuo[1], Guanwei Zhang[2], Matthew Massett[3], Jun Cheng[1], Zicong Guo[1], Liang Wang[2], Yifei Gao[2], Ru Li[2], Xu Huang[3]*, Pilong Li[2]*, and Zhi Qi[1]*

[1]Center for Quantitative Biology, Peking-Tsinghua Center for Life Sciences, Academy for Advanced Interdisciplinary Studies, Peking University, Beijing 100871, China

[2]Beijing Advanced Innovation Center for Structural Biology, Beijing Frontier Research Center for Biological Structure, Tsinghua University-Peking University Joint Center for Life Sciences, School of Life Sciences, Tsinghua University, Beijing 100084, China

[3]Paul O'Gorman Leukaemia Research Centre, Institute of Cancer Sciences, MVLS, University of Glasgow, Glasgow G12 0ZD, UK

*To whom correspondence should be addressed: zhiqi7@pku.edu.cn; pilongli@mail.tsinghua.edu.cn; xu.huang@glasgow.ac.uk

Table of contents

1. Supplementary Methods
2. Supplementary Figures
3. Supplementary Tables
4. Supplementary References

1. Supplementary Methods

Oligo Preparation

11×, 7×, 5×, 1× Gal4 binding sites and microsatellite sequence, 25×, 13×, 9×, 7×, 5×, and 3× GGAA repeats were directly ordered from Ruibiotech. The linker between Gal4 was 10-bp, and there was no linker between GGAA sequences.

DNA for *in vitro* droplet experiment were prepared by PCR from 11× vector for FUS-Gal4, and microsatellite DNA for EWS/FLI. Those PCR products were then purified by the spin column (Tiangen).

25-bp DNA substrates used for the electrophoretic mobility shift (EMSA) assay were generated by a slow annealing protocol. In the annealing system, top strand and bottom strand were added followed the molar ratio of 1:1.2 in an annealing buffer containing 40 mM Tris-HCl (pH 8.0), 50 mM NaCl, and 10 mM $MgCl_2$. Then we loaded the 1.5 ml Eppendorf tube in a 1l beaker of 800 ml water, heated it to 95°C for 5 minutes, and put it on a wood bench for a ~3 hours slow cooling to room temperature.

RNA containing 6× MS2 binding sites was transcribed from the DNA template using T7 *in vitro* transcription kit followed the instructions (TAKARA, Cat. 6140).

All oligo sequences were reported below, and the relevant DNA binding sites were highlighted in red:

(1) DNA substrates design for the *in vitro* droplet experiment

The fluorescence dye Quasar 670 was added on the PCR product by modifying one 5’ end of the forward primer with a Quasar 670 (Ruibiotech, China).

11× Gal4DBD binding sites (326-bp): 5' – CGG CCA GTG AAT TCC CAT GG – *CGG AGG ACA GTC CTC CG* – TT GCC GCC TT – *CGG AGG ACA GTC CTC CG* – AA CAA GAT GA – *CGG AGG ACA GTC CTC CG* – AG AAT CCA CT – *CGG AGG ACA GTC CTC CG* – CT GCG CAG CT – *CGG AGG ACA GTC CTC CG* – CT TCG CCA

AG – *CGG AGG ACA GTC CTC CG* – CA TGG GCT GT – *CGG AGG ACA GTC CTC CG* – AA CGT TTG TA – *CGG AGG ACA GTC CTC CG* – GA TGG ATT CG – *CGG AGG ACA GTC CTC CG* – AC AAT TTG CA – *CGG AGG ACA GTC CTC CG* – AA TGA CGG GA – *CGG AGG ACA GTC CTC CG* – CT CGA GAA GCT TGG CGT AA – 3'

9× DNA sequence (326-bp): 5'- TAA GTT GGG TAA CGC CAG GGT TTT CCC AGT CAC GAC GTT GTA AAA CGA CGG CCA GTG AAT TCG TCG AC - *CGG AGG ACA GTC CTC CG* - AG AAT CCA CT - *CGG AGG ACA GTC CTC CG* - CT GCG CAG CT - *CGG AGG ACA GTC CTC CG* - CT TCG CCA AG - *CGG AGG ACA GTC CTC CG* - CA TGG GCT GT - *CGG AGG ACA GTC CTC CG* - AA CGT TTG TA - *CGG AGG ACA GTC CTC CG* - GA TGG ATT CG - *CGG AGG ACA GTC CTC CG* - AC AAT TTG CA - *CGG AGG ACA GTC CTC CG* - AA TGA CGG GA - *CGG AGG ACA GTC CTC CG* - AC TAG TAA GCT TGG TGT AAT CAT GG -3'

7× DNA sequence (326-bp): 5'- GCT ATT ACG CCA GCT GGC GAA AGG GGG ATG TGC TGC AAG GCG ATT AAG TTG GGT AAC GCC AGG GTT TTC CCA GTC ACG ACG TTG TAA AAC GAC GGC CAG TGA ATT CGA GCT CGG TAC CGT CGA C - *CGG AGG ACA GTC CTC CG* - CTT CGC CAA G - *CGG AGG ACA GTC CTC CG* - CAT GGG CTG T - *CGG AGG ACA GTC CTC CG* - AAC GTT TGT A - *CGG AGG ACA GTC CTC CG* - GAT GGA TTC G - *CGG AGG ACA GTC CTC CG* - ACA ATT TGC A - *CGG AGG ACA GTC CTC CG* - AAT GAC GGG A - *CGG AGG ACA GTC CTC CG* - ACT AGT AAG CTT GGC GTA ATC AT -3'

5× DNA sequence (326-bp): 5'- CAT GGG CTG T - *CGG AGG ACA GTC CTC CG* - AAC GTT TGT A - *CGG AGG ACA GTC CTC CG* - GAT GGA TTC G - *CGG AGG ACA GTC CTC CG* - ACA ATT TGC A - *CGG AGG ACA GTC CTC CG* - AAT GAC GGG A - *CGG AGG ACA GTC CTC CG* - CTC GAG AAG CTT GGC GTA ATC ATG

GTC ATA GCT GTT TCC TGT GTG AAA TTG TTA TCC GCT CAC AAT TCC ACA CAA CAT ACG AGC CGG AAG CAT AAA GTG TAA AGC CTG GGG TGC CTA ATG AGT GAG CTA ACT CAC ATT AAT TGC GTT GCG CTC ACT GCC CGC TTT CCA GTC GGG AAA CCT GTC GTG CCA GC -3'

0× DNA sequence (326-bp): 5'- CTC GAG AAG CTT GGC GTA ATC ATG GTC ATA GCT GTT TCC TGT GTG AAA TTG TTA TCC GCT CAC AAT TCC ACA CAA CAT ACG AGC CGG AAG CAT AAA GTG TAA AGC CTG GGG TGC CTA ATG AGT GAG CTA ACT CAC ATT AAT TGC GTT GCG CTC ACT GCC CGC TTT CCA GTC GGG AAA CCT GTC GTG CCA GCT GCA TTA ATG AAT CGG CCA ACG CGC GGG GAG AGG CGG TTT GCG TAT TGG GCG CTC TTC CGC TTC CTC GCT CAC TGA CTC GCT GCG CTC GGT CGT TCG GCT GCG GCG AGC GGT ATC AGC TCA CTC AAA GGC GGT AA -3'

Microsatellite DNA (306-bp, 25× GGAA repeats) PCR product: 5’ – GTC GAC TAG GTT TTC CTC TTA TGC TGA GAA TTC CAG GTC CTG GAG AAG AAG AAA AAG AGA AAG AAA GAG AGA GAG AGA AGG AGT GAG AGA GGG AGG GAG GGA GGG AGG GAG GGA – *GGAA GGAA GGAA GGAA GGAA GGAA GGAA AGGAA GGAA GGAA GGAA GGAA GGAA GGAA AGGAA GGAA GGAA GGAA GGAA GGAA GGAA GGAA GGAA GGAA GGAA* – AAG AAA CAG CAA AAA AAG AAA GAG GGA GGA TGG GAG GGA GGG AAA AAG TAA AAA TGA TTC TGT ATC AGC TGG TAT ATA CCA ACA ACT AGT – 3’

(2) For Lambda DNA cloning

11× Gal4DBD binding sites (326-bp) PCR product: 5’ – CGG CCA GTG AAT TCC CAT GG – *CGG AGG ACA GTC CTC CG* – TTG CCG CCT T – *CGG AGG ACA GTC CTC CG* – AAC AAG ATG A – *CGG AGG ACA GTC CTC CG* – AGA ATC CAC T –

CGG AGG ACA GTC CTC CG – CTG CGC AGC T – *CGG AGG ACA GTC CTC CG* – CTT CGC CAA G – *CGG AGG ACA GTC CTC CG* – CAT GGG CTG T – *CGG AGG ACA GTC CTC CG* – AAC GTT TGT A – *CGG AGG ACA GTC CTC CG* – GAT GGA TTC G – *CGG AGG ACA GTC CTC CG* – ACA ATT TGC A – *CGG AGG ACA GTC CTC CG* – AAT GAC GGG A – *CGG AGG ACA GTC CTC CG* – 3’

7× Gal4DBD binding sites (222-bp) PCR product: 5’ – TCG AGC TCG GTA CCG TCG AC – *CGG AGG ACA GTC CTC CG* – CTT CGC CAA G – *CGG AGG ACA GTC CTC CG* – CAT GGG CTG T – *CGG AGG ACA GTC CTC CG* – AAC GTT TGT A – *CGG AGG ACA GTC CTC CG* – GAT GGA TTC G – *CGG AGG ACA GTC CTC CG* – ACA ATT TGC A – *CGG AGG ACA GTC CTC CG* – AAT GAC GGG A – *CGG AGG ACA GTC CTC CG* – ACT AGT AAG CTT GGC GTA ATC AT – 3’

5× Gal4DBD binding sites (152-bp) PCR product: 5’ – *CGG AGG ACA GTC CTC CG* – AAC GTT TGT A – *CGG AGG ACA GTC CTC CG* – GAT GGA TTC G – *CGG AGG ACA GTC CTC CG* – ACA ATT TGC A – *CGG AGG ACA GTC CTC CG* – AAT GAC GGG A – *CGG AGG ACA GTC CTC CG* – CTC GAG AAG CTT GGC GTA A – 3’

1× Gal4DBD binding site (162-bp) PCR product: 5’ – TAT AGT CGA C – *CGG AGG ACA GTC CTC CG* – CCG TGT AAG GGG GAT TTC TGT TCA TGG GGG TAA TGA TAC CGA TGA AAC GAG AGA GGA TGC TCA CGA TAC GGG TTA CTG ATG ATG AAC ATG CCC GGT TAC TGG AAC GTT GTG AGG GTA AAC AAC TGA CTA GTT ATA – 3’

Microsatellite DNA (306-bp, 25× GGAA repeats) PCR product: 5’ – GTC GAC TAG GTT TTC CTC TTA TGC TGA GAA TTC CAG GTC CTG GAG AAG AAG AAA AAG AGA AAG AAA GAG AGA GAG AGA AGG AGT GAG AGA GGG AGG GAG GGA GGG AGG GAG GGA – *GGAA GGAA GGAA GGAA GGAA GGAA GGAA AGGAA GGAA GGAA GGAA GGAA GGAA GGAA AGGAA GGAA GGAA GGAA GGAA*

GGAA GGAA GGAA GGAA GGAA GGAA – AAG AAA CAG CAA AAA AAG AAA GAG GGA GGA TGG GAG GGA GGG AAA AAG TAA AAA TGA TTC TGT ATC AGC TGG TAT ATA CCA ACA ACT AGT – 3’

PCR product containing 3× GGAA repeats (182-bp): 5’ – TAT AGT CGA C *GGAA GGAA GGAA* GCC ATT CGC CAT TCA GGC TGC GCA ACT GTT GGG AAG GGC GAT CGG TGC GGG CCT CTT CGC TAT TAC GCC AGC TGG CGA AA GGG GGA TGT GCT GCA AGG CGA TTA AGT TGG GTA ACG CCA GGG TTT TCC CAG TCA CGA CGT TGT AAA ACG A – 3’

PCR product containing 5× GGAA repeats (190-bp): 5’ – TAT AGT CGA C *GGAA GGAA GGAA GGAA GGAA* GCC ATT CGC CAT TCA GGC TGC GCA ACT GTT GGG AAG GGC GAT CGG TGC GGG CCT CTT CGC TAT TAC GCC AGC TGG CGA AA GGG GGA TGT GCT GCA AGG CGA TTA AGT TGG GTA ACG CCA GGG TTT TCC CAG TCA CGA CGT TGT AAA ACG A – 3’

PCR product containing 7× GGAA repeats (198-bp): 5’ – TAT AGT CGA C *GGAA GGAA GGAA GGAA GGAA GGAA GGAA* GCC ATT CGC CAT TCA GGC TGC GCA ACT GTT GGG AAG GGC GAT CGG TGC GGG CCT CTT CGC TAT TAC GCC AGC TGG CGA AA GGG GGA TGT GCT GCA AGG CGA TTA AGT TGG GTA ACG CCA GGG TTT TCC CAG TCA CGA CGT TGT AAA ACG A – 3’

PCR product containing 9× GGAA repeats (206-bp): 5’ – TAT AGT CGA C *GGAA GGAA GGAA GGAA GGAA GGAA GGAA GGAA GGAA* GCC ATT CGC CAT TCA GGC TGC GCA ACT GTT GGG AAG GGC GAT CGG TGC GGG CCT CTT CGC TAT TAC GCC AGC TGG CGA AA GGG GGA TGT GCT GCA AGG CGA TTA AGT TGG GTA ACG CCA GGG TTT TCC CAG TCA CGA CGT TGT AAA ACG A – 3’

PCR product containing 13× GGAA repeats (222-bp): 5’ – TAT AGT CGA C *GGAA GGAA GGAA GGAA GGAA GGAA GGAA GGAA GGAA GGAA GGAA GGAA GGAA*

GCC ATT CGC CAT TCA GGC TGC GCA ACT GTT GGG AAG GGC GAT CGG TGC GGG CCT CTT CGC TAT TAC GCC AGC TGG CGA AA GGG GGA TGT GCT GCA AGG CGA TTA AGT TGG GTA ACG CCA GGG TTT TCC CAG TCA CGA CGT TGT AAA ACG A – 3'

PCR product containing T7 promoter (97-bp): 5' – TAT ACT CGA C *TAA TAC GAC TCA CTA TAG G* GG ATC CTA AGG TAC CTA ATT GCC TAG AAA ACA TGA GGA TCA CCC ATG TCT GCA GGT CGA CTC TAG AAA – 3'

PCR product containing T7 promoter and 6× MS2 loop (371-bp): 5' – GC-TAA TACGACTCACTATAGG-GGA TCC TAA GGT ACC TAA TTG CCT AGA AA ACATGAGGATCACCCATGT CTG CAG GTC GAC TCT AGA AA ACATGAGGATCACCCATGT CTG CAG TAT TCC CGG GTT CAT TAG ATC CTA AGG TAC CTA ATT GCC TAG AAA ACATGAGGATCACCCATGT CTG CAG GTC GAC TCT AGA AA ACATGAGGATCACCCATGT CTG CAG TAT TCC CGG GTT CAT TAG ATC CTA AGG TAC CTA ATT GCC TAG AAA ACATGAGGATCACCCATGT CTG CAG GTC GAC TCC AGA AA ACATGAGGATCACCCATGT CTG CAG TAT TCC CGG GTT CAT TAA CTA GTG TGG CTG TGA GAC GCG – 3'

(3) For the experiments of electrophoretic mobility shift assay (see below)

1× Gal4DBD binding site Top (25-bp): 5' – ATA T – *CGG AGG ACA GTC CTC CG* – AAT A – 3'

1× Gal4DBD binding site Bottom (25-bp): 5' – TAT TCG GAG GAC TGT CCT CCG ATA T – 3'

Microsatellite DNA (306-bp): As shown in (2).

DNA template for 6× MS binding sites (375-bp): 5' – TAA TACGACTCACTATAGG-GGA TCC TAA GGT ACC TAA TTG CCT AGA AA ACATGAGGATCACCCATGT CTG

CAG GTC GAC TCT AGA AA ACATGAGGATCACCCATGT CTG CAG TAT TCC CGG GTT CAT TAG ATC CTA AGG TAC CTA ATT GCC TAG AAA ACATGAGGATCACCCATGT CTG CAG GTC GAC TCT AGA AA ACATGAGGATCACCCATGT CTG CAG TAT TCC CGG GTT CAT TAG ATC CTA AGG TAC CTA ATT GCC TAG AAA ACATGAGGATCACCCATGT CTG CAG GTC GAC TCC AGA AA ACATGAGGATCACCCATGT CTG CAG TAT TCC CGG GTT CAT TAA CTA GTG TGG CTG TGA GAC GCG – 3’

DNA template for 6× MS2 loop (350-bp): 5’ – GGA TCC TAA GGT ACC TAA TTG CCT AGA AA ACATGAGGATCACCCATGT CTG CAG GTC GAC TCT AGA AA ACATGAGGATCACCCATGT CTG CAG TAT TCC CGG GTT CAT TAG ATC CTA AGG TAC CTA ATT GCC TAG AAA ACATGAGGATCACCCATGT CTG CAG GTC GAC TCT AGA AA ACATGAGGATCACCCATGT CTG CAG TAT TCC CGG GTT CAT TAG ATC CTA AGG TAC CTA ATT GCC TAG AAA ACATGAGGATCACCCATGT CTG CAG GTC GAC TCC AGA AA ACATGAGGATCACCCATGT CTG CAG TAT TCC CGG GTT CAT TAA CTA GTG TGG CTG TGA GAC GCG – 3’

Scrambled DNA (306bp): 5’ – GCA ATG TAT GTC GTT TCA GCT AAA CGG TAT CAG CAA TGT TTA TGT AAA GAA ACA GTA AGA TAA TAC TCA ACC CGA TGT TTG AGT ACG GTC ATC ATC TGA CAC TAC AGA CTC TGG CAT CGC TGT GAA GAC GAC GCG AAA TTC AGC ATT TTC ACA AGC GTT ATC TTT TAC AAA ACC GAT CTC ACT CTC CTT TGA TGC GAA TGC CAG CGT CAG ACA TCA TAT GCA GAT ACT CAC CTG CAT CCT GAA CCC ATT GAC CTC CAA CCC CGT AAT AGC GAT GCG TAA TGA TGT CGA TAG TTA CTA ACG GGT CTT – 3’

(4) For Lambda DNA preparation

ST-forward-Bio: 5’ – (Phos) – AGG TCG CCG CCC – BIOTEG – 3’

2. Supplementary Figures

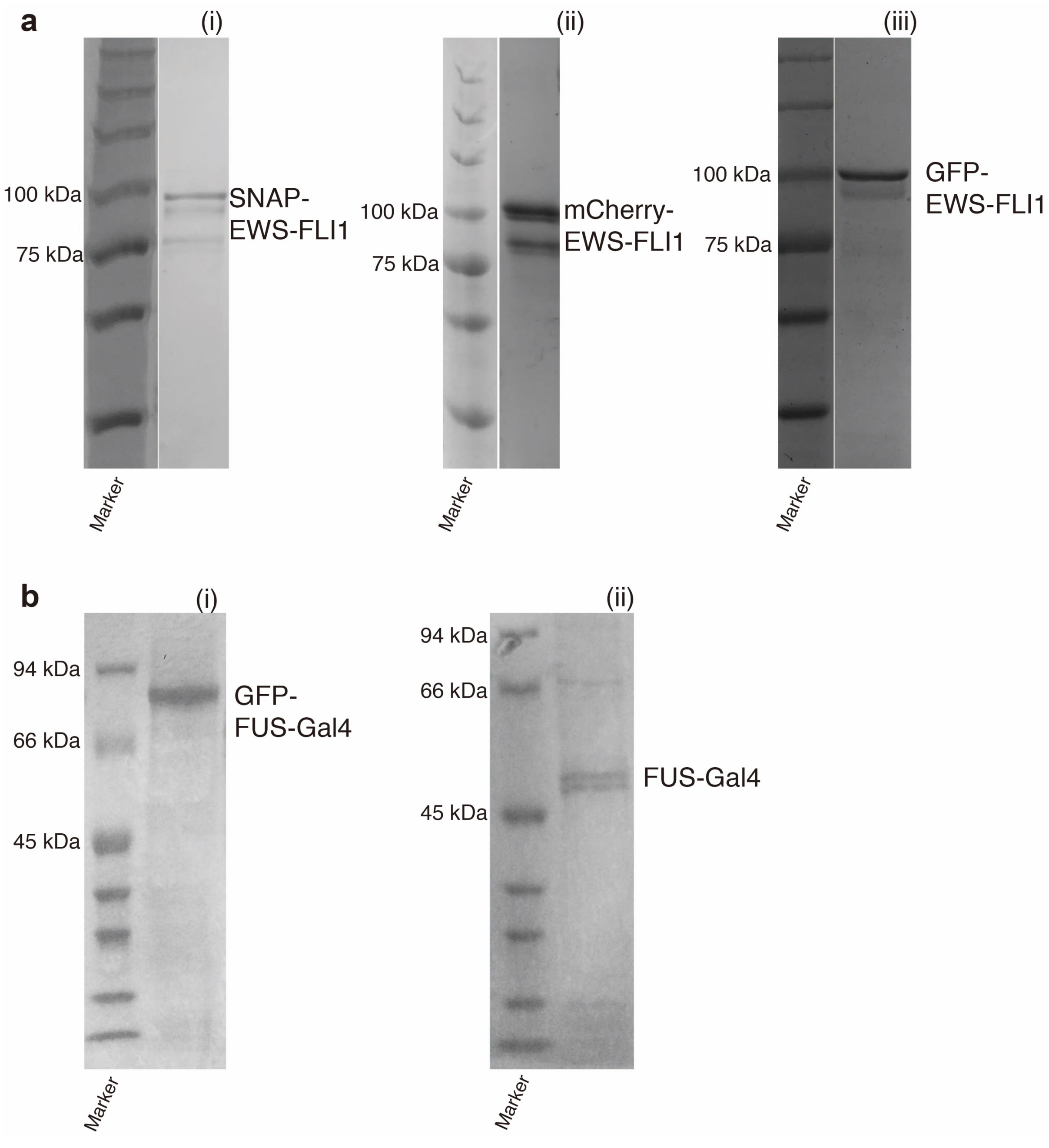

Supplementary Fig. 1 | Analysis of *in vitro* purified FET fusion proteins by SDS-PAGE. a SDS-PAGE for SNAP-EWS-FLI1 (i), mCherry-EWS-FLI1 (ii), and GFP-EWS-FLI1 (iii). **b** SDS-PAGE for GFP-FUS-Gal4 (i) and FUS-Gal4 (ii). The SDS-PAGE in a and b were repeated three times.

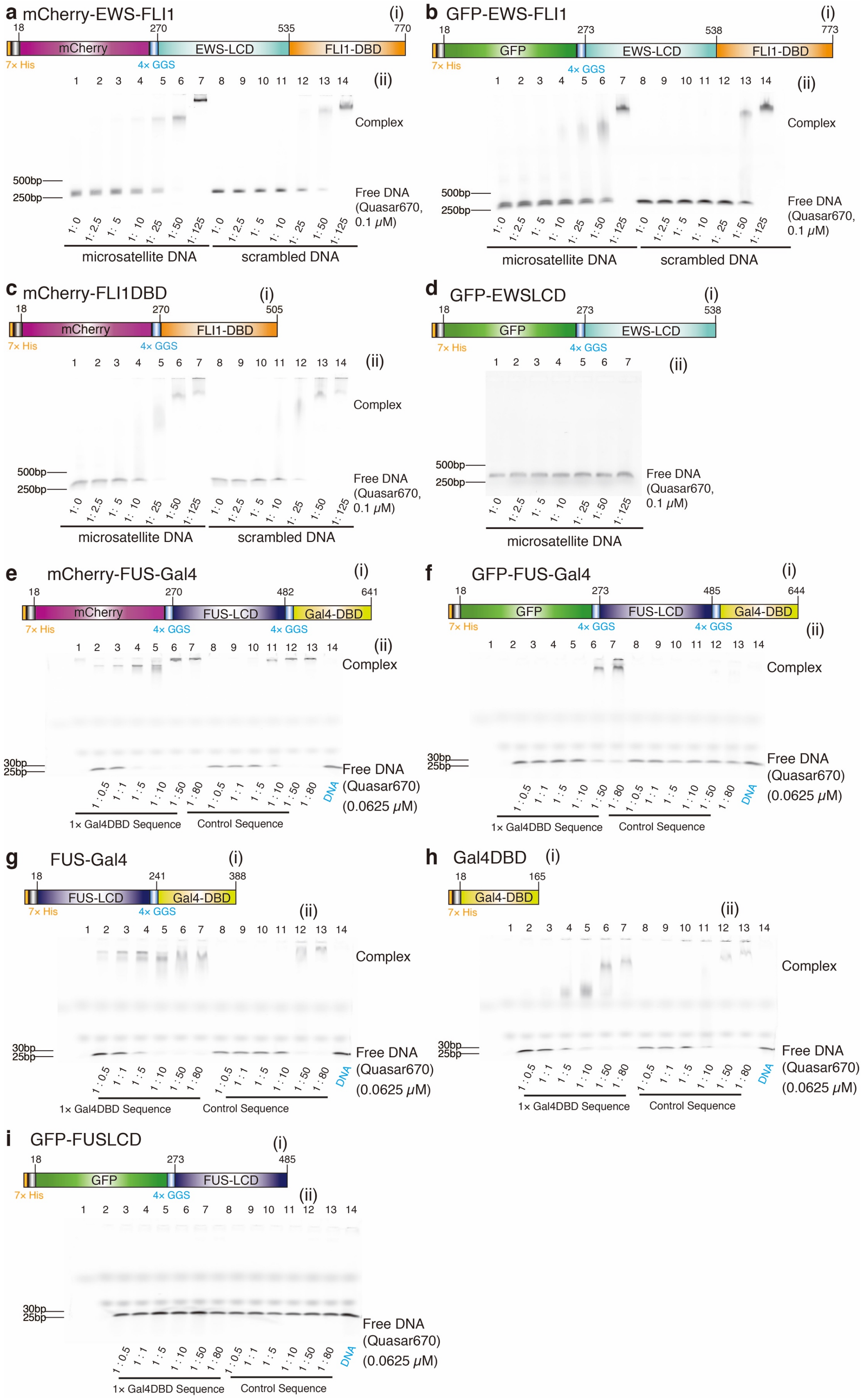

Supplementary Fig. 2 | *In vitro* purified FET fusion proteins can bind at their DNA binding motifs. a-d mCherry-EWS-FLI1 a, GFP-EWS-FLI1 b, mCherry-FLI1DBD c,

GFP-EWSLCD d. (i) Schematic; (ii) EMSA (1.2% agarose gel). We chose a 306-bp microsatellite DNA as the specific binding sequence from the previous references [1-4], and a scrambled 306-bp DNA as the controls sequence. **e-i** mCherry-FUS-Gal4 e, GFP-FUS-Gal4 f, FUS-Gal4 g, Gal4DBD h, and GFP-FUSLCD (i). (i) Schematic; (ii) EMSA (12% native PAGE gel). We chose a 25-bp DNA containing 1× Gal4DBD binding site as the specific binding sequence, and a scrambled 25-bp DNA as the controls sequence. The working buffer included 40 mM Tris-HCl (pH 7.5), 150 mM KCl, 2 mM $MgCl_2$, 1 mM DTT and 0.2 mg/ml BSA. The working buffer for EMSA was the same as the working buffer for DNA Curtains experiments. DNA substrates were labeled with Quasar670, and imaged by an Amersham Typhoon RGB (with a 635 nm laser and Cy5 670BP30 filter). All EMSAs in a-i were repeated three times.

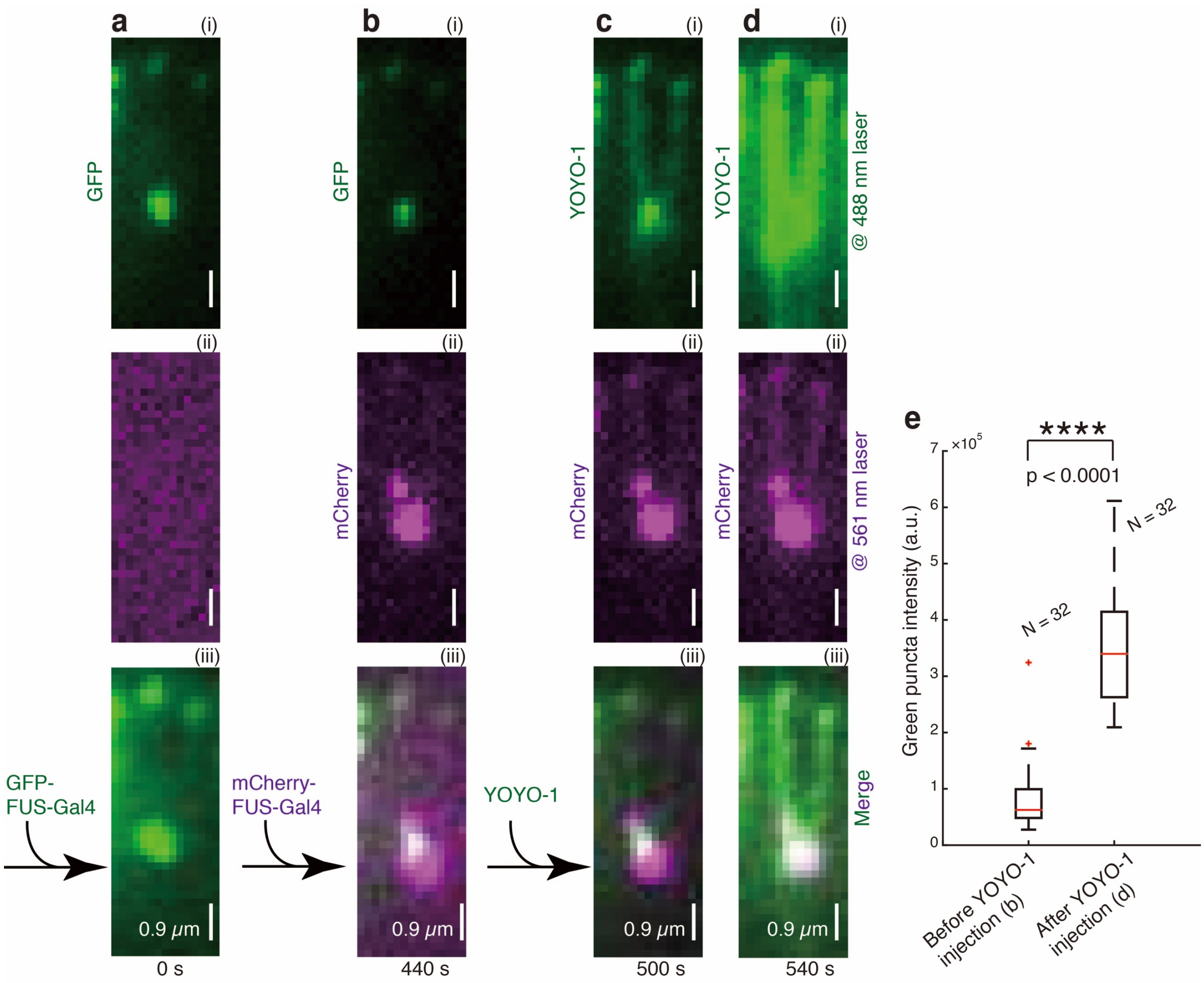

Supplementary Fig. 3 | GFP-FUS-Gal4 and mCherry-FUS-Gal4 colocalized on DNA Curtains. Supplementary Movie 2 was quantified here in three steps. **a** Step 1 (time point 0 s). 140 nM FUS-Gal4 mixed with 10 nM GFP-FUS-Gal4 was injected into the flow cell with a flow rate of 0.4 ml/min for 6 minutes. A green punctum was formed on Lambda DNA containing 11× Gal4DBD binding sites during this process; **b** Step 2 (time point 440 s). Afterward, 50 nM mCherry-FUS-Gal4 was injected into the flow cell with a flow rate of 0.4 ml/min for 6 minutes. A magenta punctum was colocalized with the green punctum; **c-d** Step 3 (time point 500 s and 540 s). a working buffer with 0.5 nM YOYO1 was used to wash out all free mCherry-FUS-Gal4 from the chamber, and DNA molecules were stained with YOYO1. (i) the 488 nm laser channel; (ii) the 561 nm laser channel; (iii) Two channels were merged. **e** Boxplot of the green puncta

intensity before b(i) and after d(i) YOYO-1injection. The total number N of the green puncta examined over one time DNA Curtains experiment: N = 32 before b(i), N = 32 after d(i). For the boxplot, the red bar represents median. The bottom edge of the box represents 25th percentiles, and the top is 75th percentiles. Most extreme data points are covered by the whiskers except outliers. The '+' symbol is used to represent the outliers. Statistical significance was analyzed using unpaired t test for two groups. p value: two-tailed; p value style: GP: 0.1234 (ns), 0.0332 (*), 0.0021 (**), 0.0002 (***), <0.0001 (****). Confidence level: 95%.

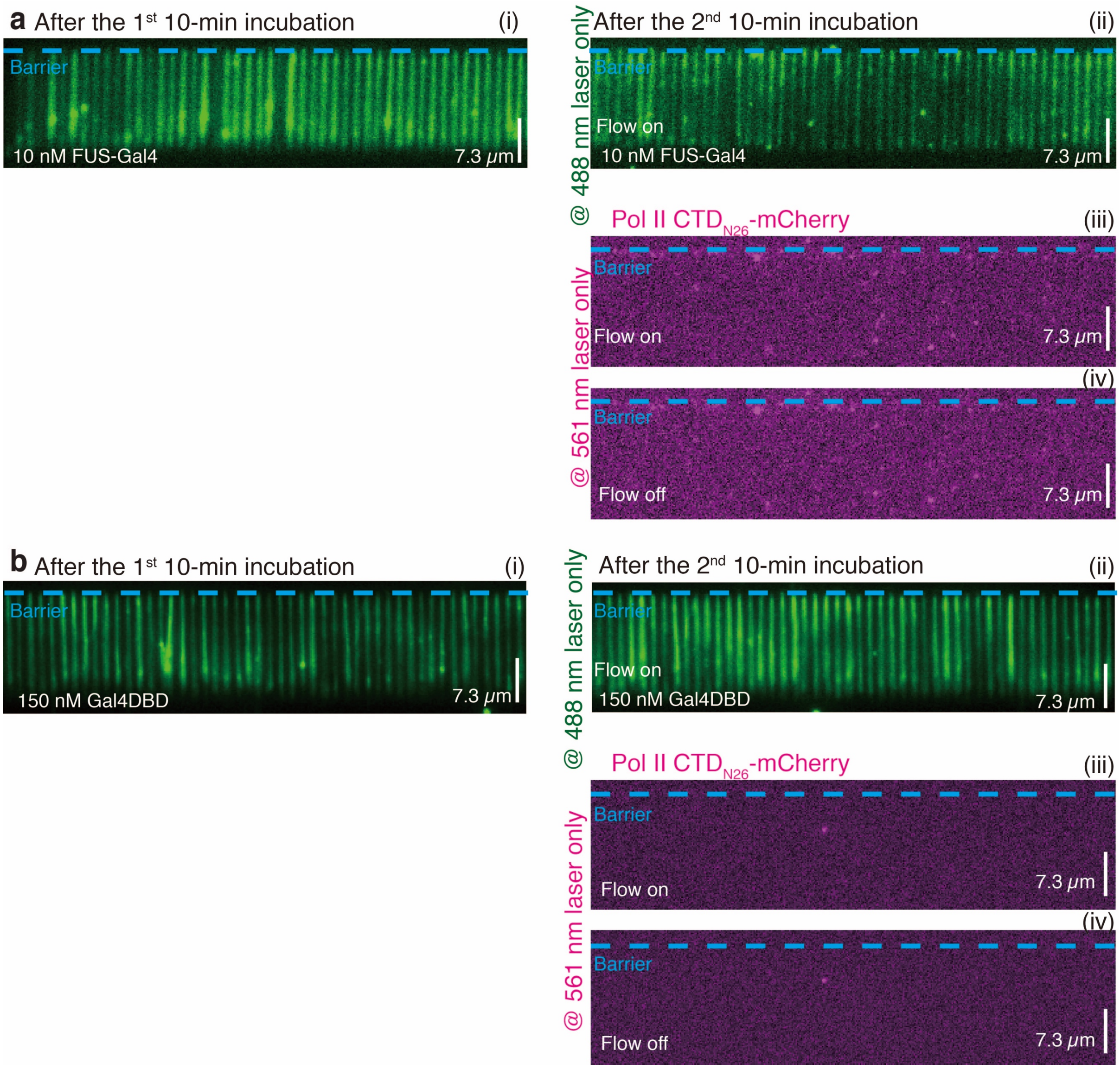

Supplementary Fig. 4 | Pol II CTD cannot be recruited at DNA binding motifs if no FET fusion protein condensates. a 10 nM FUS-Gal4 or **b** 150 nM Gal4DBD was used to repeat DNA curtains experiments in Fig. 3b(i) and h. (i) Wide-field TIRFM images of DNA Curtains after the 1st 10-min incubation, like in Fig. 3b(i). (ii)-(iv) Wide-field TIRFM images of DNA Curtains after the 2nd 10-min incubation, like Fig. 3h. (ii) only the 488-nm laser on and flow on; (iii)-(iv) only the 561-nm laser on and flow on (iii) / off (iv). The DNA Curtains experiments in a and b were repeated three times.

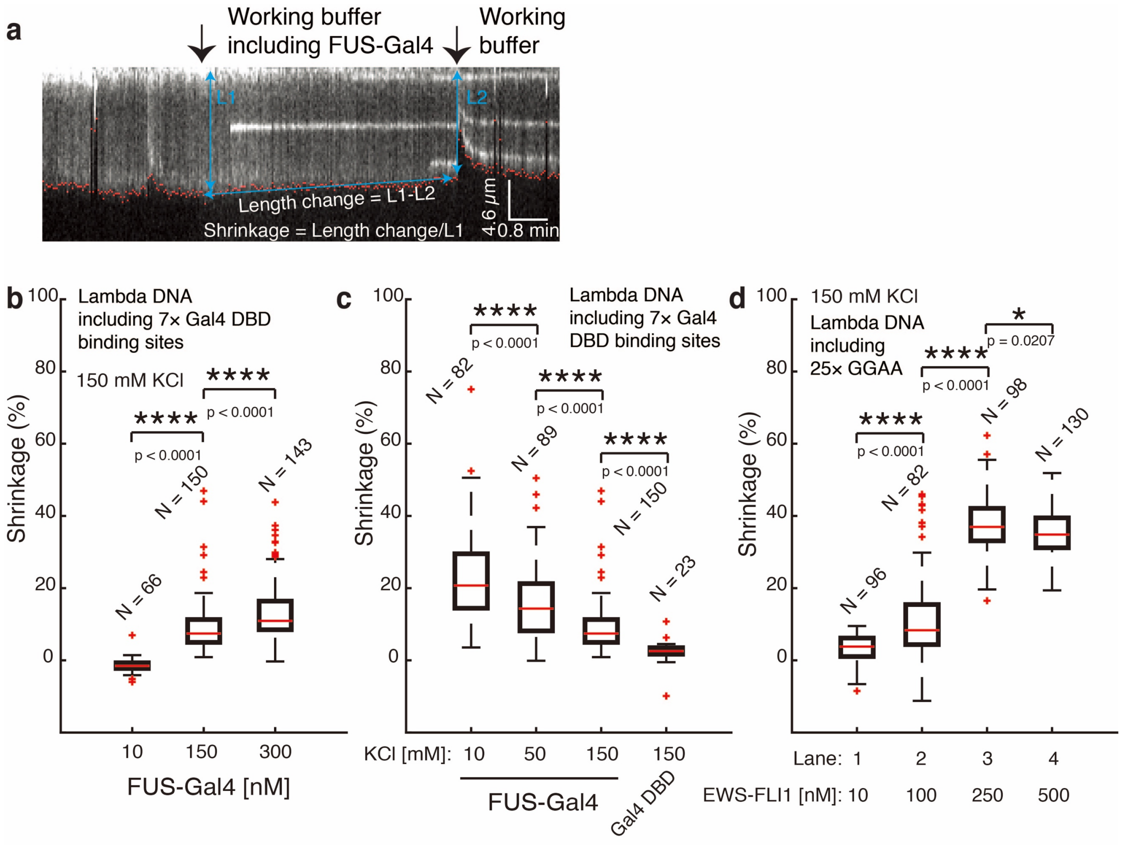

Supplementary Fig. 5 | FET fusion protein condensates slightly compacted DNA *in vitro*. a A representative kymograph showing the image tracking of DNA end (red dot). L1 was the initial DNA length before FET fusion protein injection, and L2 was the DNA length after 5-min working buffer washing. Shrinkage (%) = (L1 − L2) / L1. **b-c** The influence of FUS-Gal4 concentration b and the salt concentration c on DNA length. **d** The influence of EWS-FLI1 concentration on DNA length. N was the total number of the kymographs in a examined over three times independent DNA Curtains experiments. For the boxplot, the red bar represents median. The bottom edge of the box represents 25th percentiles, and the top is 75th percentiles. Most extreme data points are covered by the whiskers except outliers. The '+' symbol is used to represent the outliers. Statistical significance was analyzed using unpaired t test for two groups. p value: two-tailed; p value style: GP: 0.1234 (ns), 0.0332 (*), 0.0021 (**), 0.0002 (***), <0.0001 (****). Confidence level: 95%.

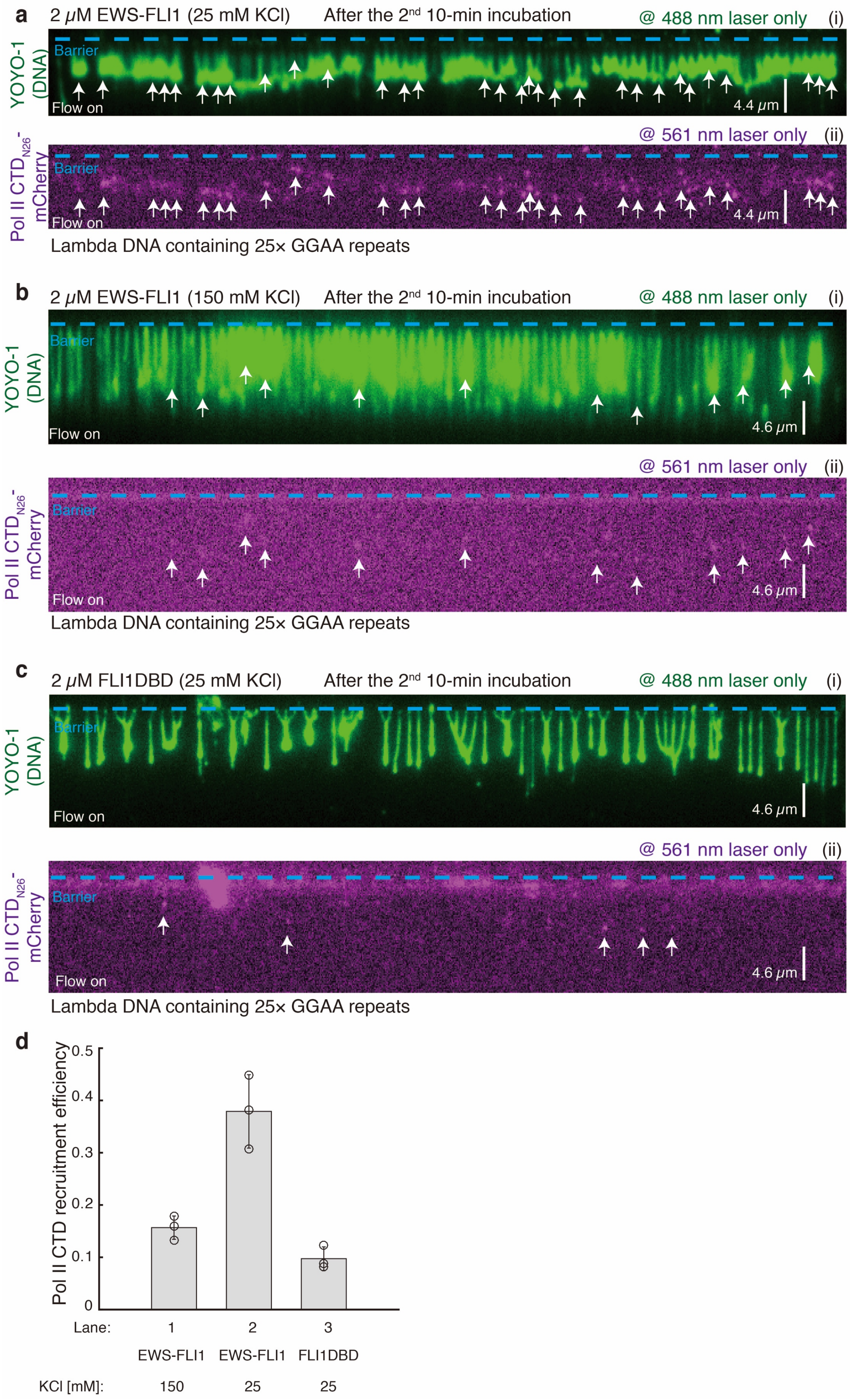

Supplementary Fig. 6 | EWS-FLI1 condensates can recruit Pol II CTD to the microsatellite loci *in vitro*. a-b Wide-field TIRFM images of EWS-FLI1 condensates

and Pol II CTD_{N26}-mCherry after the 2nd 10-min incubation (Supplementary Movie 5), like Fig. 3h. (i) only the 488-nm laser on and flow on; (ii) only the 561-nm laser on and flow on; White Arrows pointed to puncta of Pol II CTD_{N26}-mCherry colocalized with EWS-FLI1 condensates. The working buffer contained 25 mM KCl a and 150 mM KCl b. 2 μM EWS-FLI1 was used. **c** The control experiment of (a), and 2 μM FLI1DBD was used. DNA substrates were Lambda DNA containing 25× GGAA repeats. The working buffer for DNA Curtains was 40 mM Tris-HCl (pH 7.5), 25 (or 150) mM KCl, and 2 mM $MgCl_2$, 1 mM DTT, and 0.2 mg/ml BSA. **d** The efficiency of Pol II CTD_{N26}-mCherry recruitment. Lane 1, a; Lane 2, b; Lane 3, c. Independent DNA Curtains experiments were repeated: n=3 for a (Lane 1), n=3 for b (Lane 2), and n=3 for c (Lane 3). Error bars, mean ± s.d.

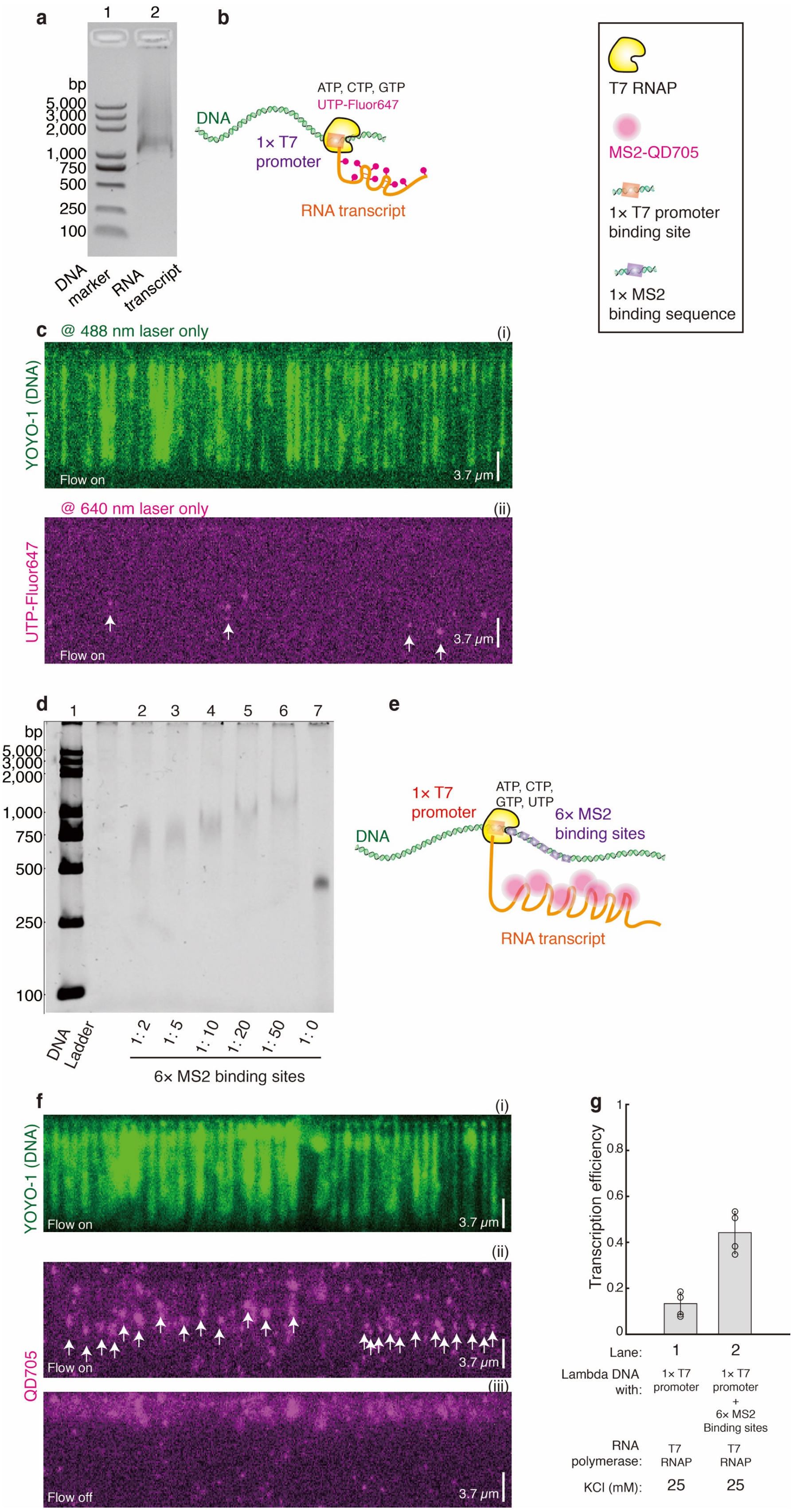

Supplementary Fig. 7 | *In vitro* transcription activity of T7 RNAP on DNA Curtains.

a Biochemistry test for T7 RNAP. Lane 1, DNA marker (GenStar, Cat. M121-01); Lane

2, RNA transcript. T7 RNAP (TAKARA, Cat. 6140) was mixed with NTP and template DNA containing 1× T7 promoter at 42°C for 2 hours. Afterward, RNase free DNase I (TAKARA, Cat. 6140) was used to digest DNA. The reaction products were loaded on a 2% agarose gel. This biochemistry test was repeated three times. **b** Schematic of the *in vitro* transcription assay on DNA Curtains. T7 RNAP and other components (ATP, CTP, GTP, UTP-Fluor647) were injected in the chamber for 20-min incubation. DNA substrates were Lambda DNA containing 1× T7 promoter. **c** Wide-field TIRFM images of nascent RNA transcripts (ii) on DNA (i) after incubation. White arrows in (ii) confirmed the labeled punctum was on DNA. **d** EMSA for MS2 bacteriophage coat protein (MCP). The EMSAs were repeated three times. RNA molecules (200 nM, 355-nt) containing 6× MS2 binding sites were pre-incubated with 3×Flag tag labeled MCP at room temperature for 30-min. The molar ratio of [RNA]:[MS2] changed from 1:2 (lane 2), 1:5 (lane 3), 1:10 (lane 4), 1:20 (lane 5), 1:50 (lane 6), and 1:0 (lane 7). The working buffer was 40 mM Tris-HCl (pH=7.5), 2 mM $MgCl_2$, 1 mM DTT, and 0.2 mg/ml BSA. The samples were loaded on a 4.5% TBE-PAGE gel. **e-f** Like (b and c). DNA substrates were Lambda DNA containing 1× T7 promoter and 6× MS2 binding sequence. T7 RNAP and other components (5 mM NTPs, no UTP-Fluor647) were injected in the chamber for 20-min incubation. Finally QD 705 labeled MS2 was injected into the chamber to label RNA transcripts. White arrows in F(ii) confirmed the labeled punctum was on DNA. **g** Transcription efficiency for experimental conditions. Lane 1, c(ii); Lane 2, f(ii). Independent DNA Curtains experiments were repeated: n = 5 for c(ii), and n = 4 for f(ii). Error bars, mean ± s.d.

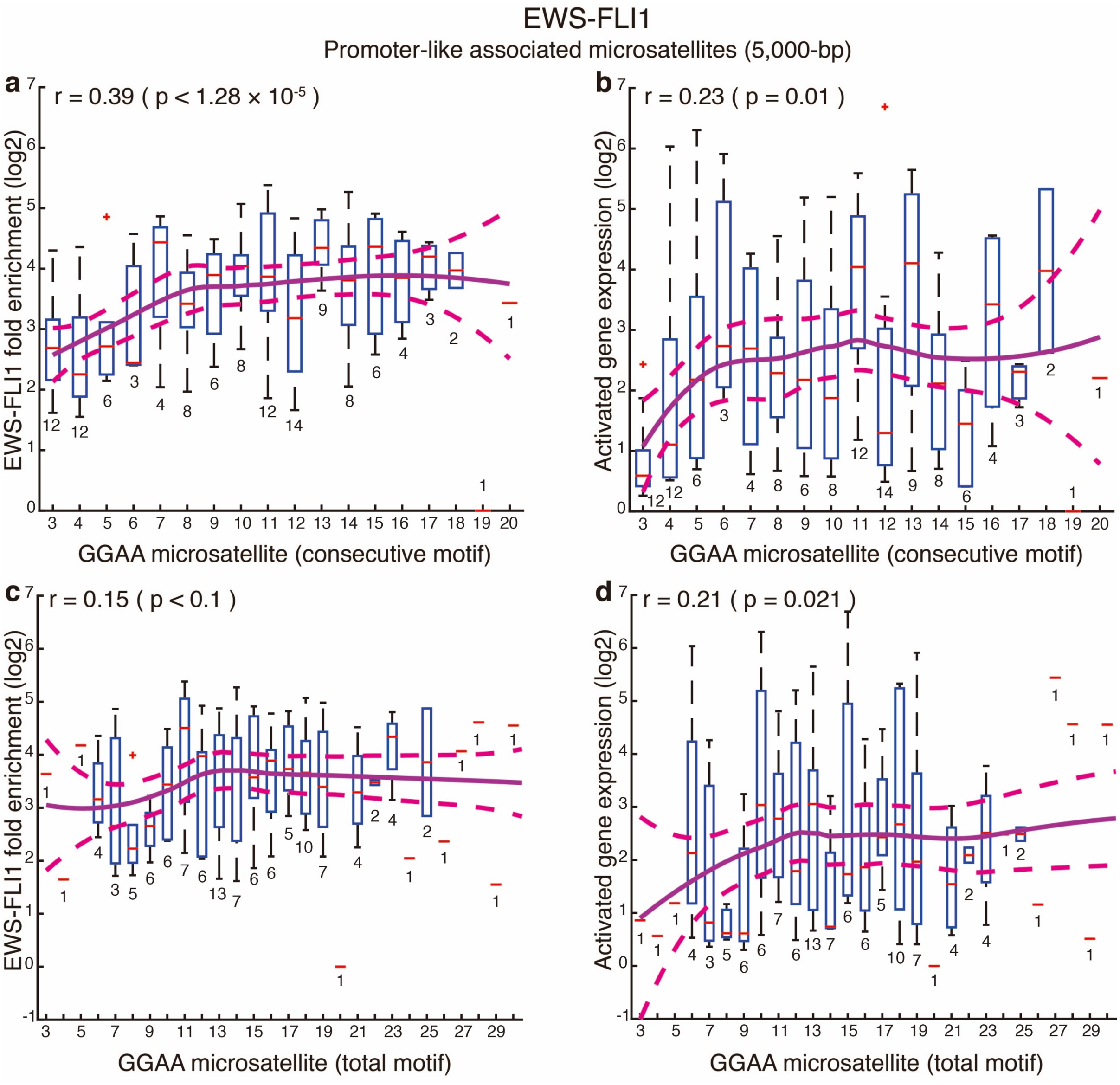

Supplementary Fig. 8 | The number of GGAA microsatellites is highly associated with the gene transcriptional regulation by FET fusion proteins. a Boxplot of EWS-FLI1 fold enrichment (relative to the input) against consecutive motif in promoter-like EWS-FLI1-bound microsatellites (5,000-bp, Methods), showing statistically significant correlation ($r = 0.39$, $p < 1.28 \times 10^{-5}$). **b** Boxplot of EWS-FLI1 activated gene expression against consecutive motif at promoter-like EWS-FLI1-bound microsatellites (5,000-bp) for gene activation, showing a non-linear trend showing a statistically significant correlation ($r = 0.23$, $p = 0.01$). **c** Boxplot of EWS-FLI1 fold enrichment (relative to the input) against total motifs in promoter-like EWS-FLI1-bound

microsatellites (5,000-bp), showing statistically significant correlation ($r = 0.15$, $p < 0.1$). **d** Boxplot of EWS-FLI1 activated gene expression against total motif at promoter-like EWS-FLI1-bound microsatellites (5,000-bp) for gene activation, showing a non-linear trend showing a statistically significant correlation ($r = 0.21$, $p = 0.021$). All correlations were calculated using Spearman's rank correlation. The purple line is a LOESS regression line with estimated 95% confidence intervals shown (the region between two dash lines). ChIP-Seq analysis was based on NCBI data (GEO: GSE99959) [2]. The total gene number for each motif of GGAA microsatellite examined in the ChIP-Seq analysis was indicated in all boxplots. For the boxplot, the red bar represents median. The bottom edge of the box represents 25th percentiles, and the top is 75th percentiles. Most extreme data points are covered by the whiskers except outliers. The '+' symbol is used to represent the outliers.

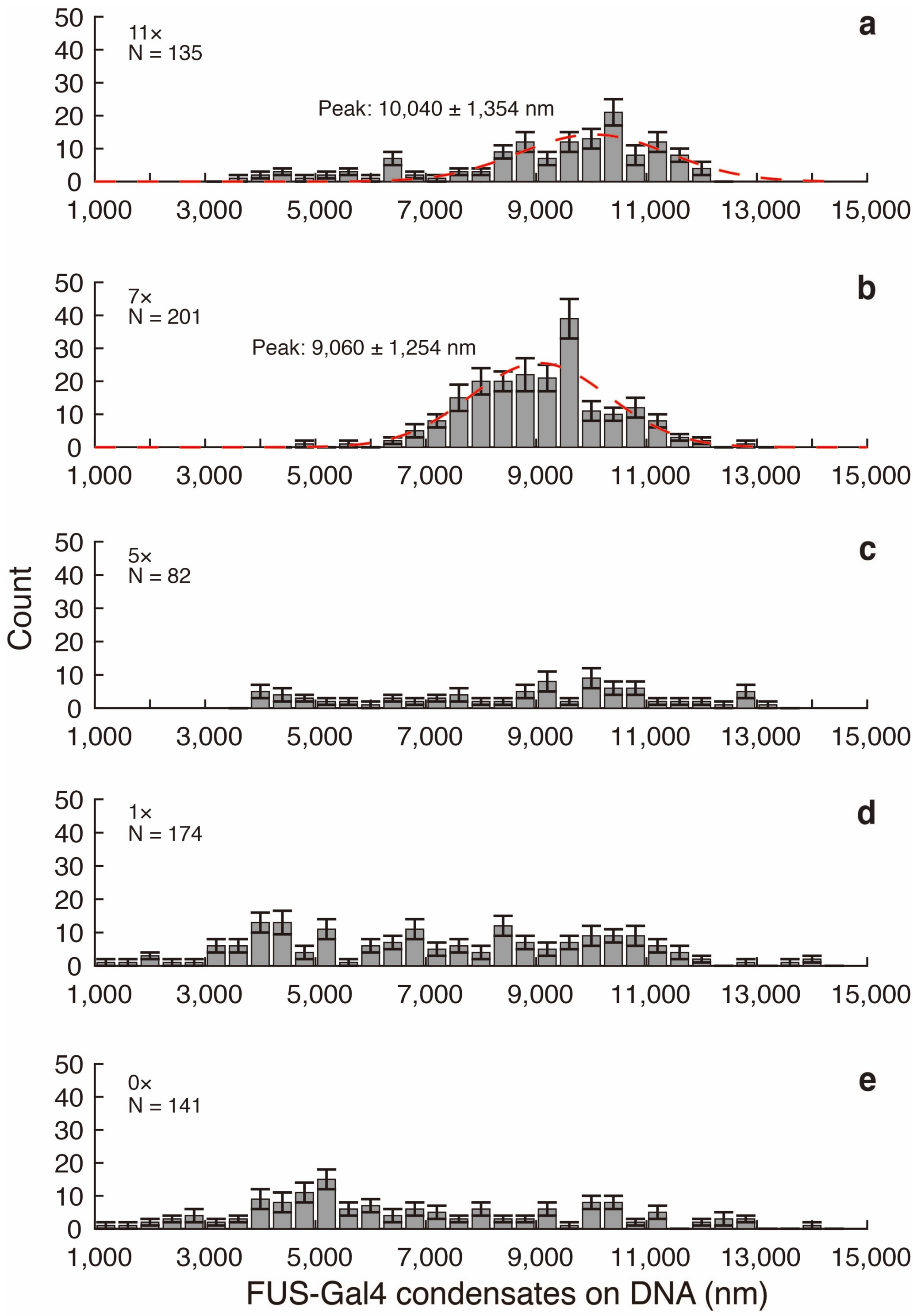

Supplementary Fig. 9 | Gal4DBD binding sites beyond a threshold drive FUS-Gal4 condensates formation. a-e Position distribution of FUS-Gal4 condensates on 11× a, 7× b, 5× c, 1× d, and 0× e Gal4DBD binding sites. 400-nm bin. The experimental procedures were in Fig. 3a(i)-(iv). The total colocalized puncta number N examined over three times DNA Curtains experiments (n = 3): N = 135 for a, N = 201 for b, N =

82 for c, N = 174 for d, and N = 141 for e. Error bars in b(ii) and h(v) were obtained through the bootstrap analysis. For any normally distributed dataset 68.27% of the values lie within one standard deviation of the mean, therefore our choice of 70% confidence intervals for the bootstrapped data provides a close approximation to expectations for one standard deviation from the mean. b was the same data as Fig. 3b(ii). a, b, and c were fitted with a 1D Gaussian function. The theoretical insertion position on Lambda DNA is 9,124 nm. The fitted peak positions in (a) and (b) are 10,040 $\pm$ 1,354 nm and 9,060 $\pm$ 1,254 nm. Error bars of the fitting parameters represent 95% confidence intervals obtained through Gaussian function fitting.

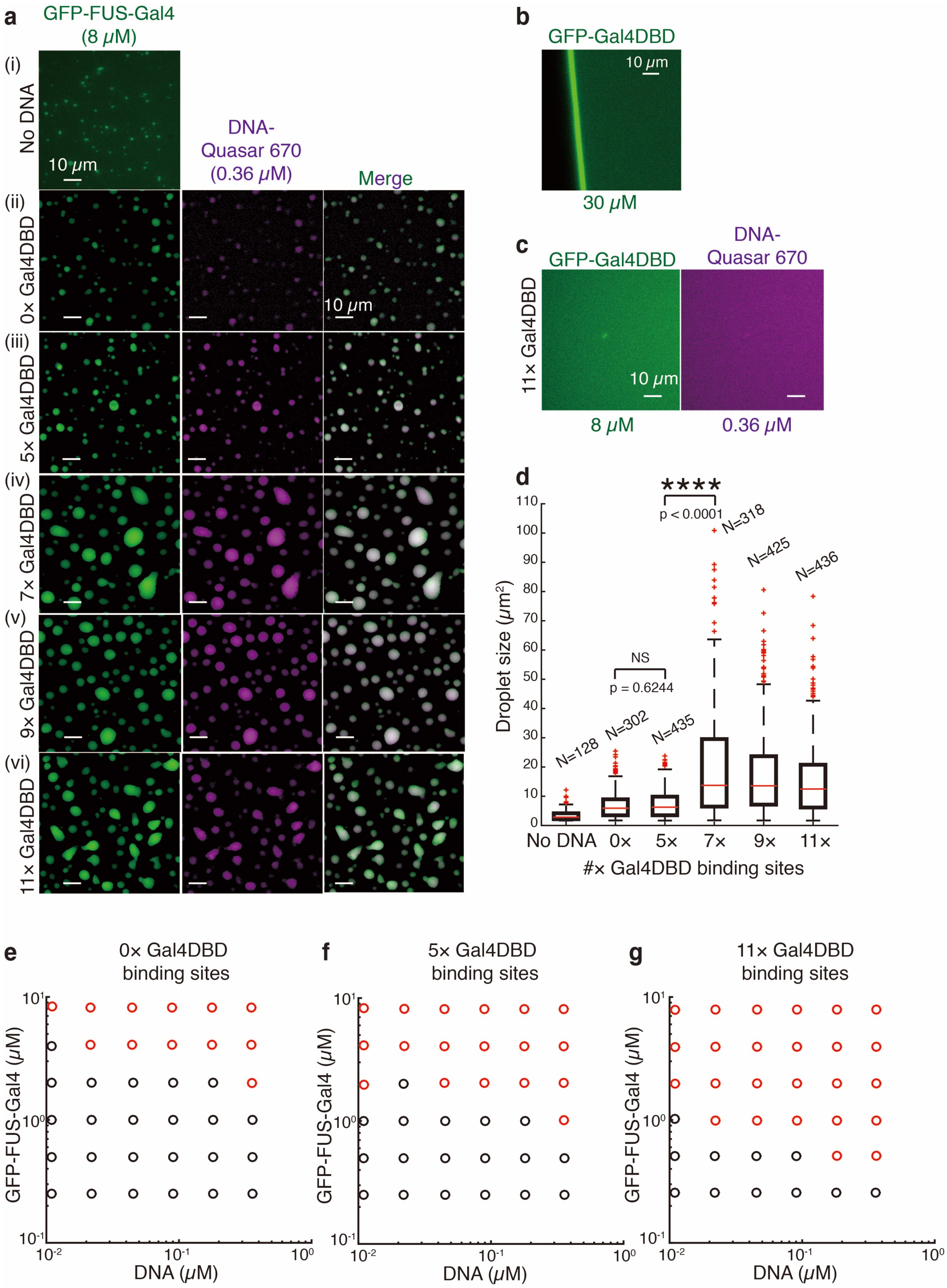

Supplementary Fig. 10 | DNA binding motifs modulate FUS-Gal4 condensate formation. a *In vitro* droplet experiments. 8 μM GFP-FUS-Gal4 was mixed without (i)

and with 0.36 μM 326-bp dsDNA, containing 0× (ii), 5× (iii), 7× (iv), or 11× (v) Gal4DBD binding sites. **b** *In vitro* droplet experiment for GFP-Gal4DBD (30 μM) alone. **c** *In vitro* droplet experiment. GFP-Gal4DBD (8 μM) was mixed with 0.36 μM 326-bp dsDNA containing 11× Gal4DBD binding sites. The *in vitro* droplet assays in a-c were repeated three times. **d** Boxplot of droplet size from a. Statistical significances were evaluated based on the student's t-test. N was the total droplet events examined over three time independent *in vitro* droplet assays. For the boxplot, the red bar represents median. The bottom edge of the box represents 25th percentiles, and the top is 75th percentiles. Most extreme data points are covered by the whiskers except outliers. The '+' symbol is used to represent the outliers. Statistical significance was analyzed using unpaired t test for two groups. p value: two-tailed; p value style: GP: 0.1234 (ns), 0.0332 (*), 0.0021 (**), 0.0002 (***), <0.0001 (****). Confidence level: 95%. **e-g** Phase separation diagram of GFP-FUS-Gal4 was mixed with 326-bp dsDNA including 0× c, 5× d, or 11× e Gal4 binding sites. Black/red dots: no/with phase separation.

3. Supplementary Tables

Supplementary Table 1. All primer sequences in this work.

Primer	Sequence (5' → 3')
GFP-FUS-Gal4-F	GTGGTAGCGGCGGCTCCATGGCCTCAAAC GATTATACCCA
FUS-Gal4-R	GGTTTCTTTACCAGACTCGAGTTACGATAC AGTCAACTGTCTTTG
FUS-Gal4-F	AACCTGTATTTTCAGGGCGCCATGGCCTCA AACGATTCTACC
Gal4 DBD-F	AACCTGTATTTTCAGGGCGCCATGAAGCTA CTGTCTTCTATC
mCherry-FUS-Gal4-F	GTGGTAGCGGCGGCTCCATGGGAATGGCC TCAAACGATTATACCCA
FUS LCD-R	CGGTTTCTTTACCAGACTCGAGTTAGTCCT GCTGTCCATAGCCAC
mCherry-FLI1 DBD-F	GTGGTAGCGGCGGCTCCATGGGACCTTCT TATGACTCAGTCAG
FLI1 DBD-R	GGTTTCTTTACCAGACTCGAGCTAGTAGTA GCTGCCTAAGTG
GFP-EWS LCD-F	GTGGTAGCGGCGGCTCCATGGCTTCCACC GACTATTC
GFP-EWS LCD-R	GGTTTCTTTACCAGACTCGAGCTGCTGACC GTAGCTAGAGG
mCherry-EWS LCD-F	GTGGTAGCGGCGGCTCCATGGGAATGGCT TCCACCGACTATTC

4. Supplementary References

1 Johnson, K. M. *et al.* Role for the EWS domain of EWS/FLI in binding GGAA-microsatellites required for Ewing sarcoma anchorage independent growth. *Proc. Natl. Acad. Sci. U. S. A.* **114**, 9870-9875, doi:10.1073/pnas.1701872114 (2017).

2 Johnson, K. M., Taslim, C., Saund, R. S. & Lessnick, S. L. Identification of two types of GGAA-microsatellites and their roles in EWS/FLI binding and gene regulation in Ewing sarcoma. *PLoS One* **12**, doi:ARTN e0186275, 10.1371/journal.pone.0186275 (2017).

3 Monument, M. J. *et al.* Clinical and Biochemical Function of Polymorphic NR0B1 GGAA-Microsatellites in Ewing Sarcoma: A Report from the Children's Oncology Group. *PLoS One* **9**, doi:ARTN e104378, 10.1371/journal.pone.0104378 (2014).

4 Gangwal, K. *et al.* Microsatellites as EWIS/FLI response elements in Ewing's sarcoma. *Proc. Natl. Acad. Sci. U. S. A.* **105**, 10149-10154, doi:10.1073/pnas.0801073105 (2008).
