# Supplementary material for: Loci-specific phase separation of FET fusion oncoproteins promotes gene transcription
Source: Nat Commun. 2021 Mar 5;12:1491. doi: 10.1038/s41467-021-21690-7 (PMC7935978; doi:10.1038/s41467-021-21690-7)
Supplement: Supplementary file 10 — Description of additional supplementary files [file 41467_2021_21690_MOESM10_ESM.docx]

Description of Additional Supplementary Information

Title: Supplementary Movie 1

Description: EWS-FLI1 molecules form biomolecular condensates at 25´ GGAA repeats. 100 nM mCherry-EWS-FLI1 were injected into a chamber of DNA Curtains. DNA substrates containing 25´ GGAA repeats were stained with YOYO1. The flow rate was 0.4 ml/min.

Title: Supplementary Movie 2

Description: GFP-FUS-Gal4 and mCherry-FUS-Gal4 colocalize on DNA Curtains. 140 nM FUS-Gal4 mixed with 10 nM GFP-FUS-Gal4 was injected into the flow cell with a flow rate of 0.4 ml/min for 6 minutes. A green punctum was formed on Lambda DNA containing 11 × Gal4DBD binding sites during this process (Supplementary Fig. 3a). Afterward, 50 nM mCherry-FUS-Gal4 was injected into the flow cell with a flow rate of 0.4 ml/min for 6 minutes. A magenta punctum was colocalized with the green punctum (Supplementary Fig. 3b). Finally, a working buffer with 0.5 nM YOYO1 was used to wash out all free mCherry-FUS-Gal4 from the chamber, and DNA molecules were stained with YOYO1 (Supplementary Fig. 3c-d). 2-s shutter time was used in this movie.

Title: Supplementary Movie 3

Description: FUS-Gal4 condensates on different DNA substrates fuse together.

Title: Supplementary Movie 4

Description: FUS-Gal4 condensates can recruit Pol II CTDN26-mCherry. After FUS-Gal4 condensates formed on the 7´ Gal4DBD binding sites, 1 μM Pol II CTDN26-mCherry was injected into the flow cell for 10-minute incubation. Afterward, we acquired the movie to show that enriched mCherry signals representing Pol II CTDN26 were detected within FUS-Gal4 condensates (Fig. 3h). (Top) only the 488-nm laser was turned on; (Bottom) only the 561-nm laser was turned on.

Title: Supplementary Movie 5

Description: EWS-FLI1 condensates can recruit Pol II CTDN26-mCherry. After EWS-FLI1 condensates formed on the 25´ GGAA repeats, 1 μM Pol II CTDN26-mCherry was injected into the flow cell for 10-minute incubation. Afterward, we acquired the movie. (Top) only the 488-nm laser was turned on; (Bottom) only the 561-nm laser was turned on. 25 mM KCl was used.

Title: Supplementary Movie 6

Description: MS2 recognizes its target RNA in vitro transcribed on DNA Curtains. We cloned 1´ T7 promoter and 6´ MS2 binding sequences into Lambda DNA. We repeated the experiment in Supplementary Fig. 7b-c with 5 mM NTPs (no UTPFluor647), and finally injected Quantum dot (QD) 705 labeled MS2 to image RNA transcripts (Supplementary Fig. 7e-f).

Title: Supplementary Movie 7

Description: Loci-specific Pol II CTD recruitment of FUS-Gal4 condensates enhances gene transcription. After FUSGal4 condensates formed on the 7´ Gal4DBD binding sites, Pol II CTDN26-T7 RNAP, Pol II CTDN26-mCherry, and NTPs (ATP, CTP, GTP, and UTP-Fluor647) were injected into the flow cell for 20-minute incubation. Afterward, we acquired the movie to show that enriched Fluor647 signals representing nascent RNA transcripts were detected within FUS-Gal4 condensates (Fig. 4a). (Top) only the 488-nm laser was turned on; (Bottom) only the 640-nm laser was turned on.
