# Supplementary material for: Loci-specific phase separation of FET fusion oncoproteins promotes gene transcription
Source: Nat Commun. 2021 Mar 5;12:1491. doi: 10.1038/s41467-021-21690-7 (PMC7935978; doi:10.1038/s41467-021-21690-7)
Supplement: Supplementary file 11 — Reporting Summary [file 41467_2021_21690_MOESM11_ESM.pdf]

nature research

Corresponding author(s): Zhi Qi

Last updated by author(s): Feb 3, 2021

Reporting Summary

Nature Research wishes to improve the reproducibility of the work that we publish. This form provides structure for consistency and transparency in reporting. For further information on Nature Research policies, see our Editorial Policies and the Editorial Policy Checklist.

Statistics

For all statistical analyses, confirm that the following items are present in the figure legend, table legend, main text, or Methods section.

n/a	Confirmed	
☐	☒	The exact sample size (*n*) for each experimental group/condition, given as a discrete number and unit of measurement
☐	☒	A statement on whether measurements were taken from distinct samples or whether the same sample was measured repeatedly
☐	☒	The statistical test(s) used AND whether they are one- or two-sided<br>*Only common tests should be described solely by name; describe more complex techniques in the Methods section.*
☒	☐	A description of all covariates tested
☐	☒	A description of any assumptions or corrections, such as tests of normality and adjustment for multiple comparisons
☐	☒	A full description of the statistical parameters including central tendency (e.g. means) or other basic estimates (e.g. regression coefficient) AND variation (e.g. standard deviation) or associated estimates of uncertainty (e.g. confidence intervals)
☐	☒	For null hypothesis testing, the test statistic (e.g. *F*, *t*, *r*) with confidence intervals, effect sizes, degrees of freedom and *P* value noted<br>*Give P values as exact values whenever suitable.*
☐	☒	For Bayesian analysis, information on the choice of priors and Markov chain Monte Carlo settings
☒	☐	For hierarchical and complex designs, identification of the appropriate level for tests and full reporting of outcomes
☐	☒	Estimates of effect sizes (e.g. Cohen's *d*, Pearson's *r*), indicating how they were calculated

Our web collection on statistics for biologists contains articles on many of the points above.

Software and code

Policy information about availability of computer code

Data collection

1. All experimental data of DNA Curtains were acquired with a custom-built prism-type total internal reflection fluorescence microscope (TIRFM) (Nikon, Inverted Microscope Eclipse Ti-E). The software was CellVision Coolight Technology Version 1.4.0 (Home-made software).
2. Luciferase assays were collected by Gene5 (Software features for imaging & microscopy). The hardware was BioTek Cytation5 (BioTek Instruments Inc.).

Data analysis

1. Image analysis was performed using Open source image processing software ImageJ (Version: 2.0.0-rc-59/1.51k, http://imagej.net/Contributors).
2. Confocal microscopy data were collected and analyzed by Nikon Elements.
3. Consecutive motif and total motif analysis was conducted by MATLAB 2016b software (https://www.mathworks.com/products/matlab.html).
4. All the R packages used for bioinformatics analysis could be downloaded from https://bioconductor.org/packages/3.12/bioc/. (1) Kallisto (https://pachterlab.github.io/kallisto/about.html); (2) FastQC (Version 0.11.8, https://www.bioinformatics.babraham.ac.uk/projects/fastqc/); (3) Trim Galore (Version 0.4.3, https://www.bioinformatics.babraham.ac.uk/projects/ trim_galore/); (4) Bowtie2 (Version 2.3.4, https://sourceforge.net/projects/bowtie-bio/files/bowtie2/2.3.4/); (5) SAMtools (Version 1.3.1, http://www.htslib.org/download/); (6) MACS2 (Version 3.4, https://pypi.org/project/MACS2/).

For manuscripts utilizing custom algorithms or software that are central to the research but not yet described in published literature, software must be made available to editors and reviewers. We strongly encourage code deposition in a community repository (e.g. GitHub). See the Nature Research guidelines for submitting code & software for further information.

Data

Policy information about availability of data

All manuscripts must include a data availability statement. This statement should provide the following information, where applicable:

- Accession codes, unique identifiers, or web links for publicly available datasets
- A list of figures that have associated raw data
- A description of any restrictions on data availability

The data that support the findings of this study are available from the corresponding author upon reasonable request. The source data underlying Figs 1-6, and Supplementary Figs 1, 3, and 5-10 are provided as a Source Data file.

Field-specific reporting

Please select the one below that is the best fit for your research. If you are not sure, read the appropriate sections before making your selection.

☒ Life sciences ☐ Behavioural & social sciences ☐ Ecological, evolutionary & environmental sciences

For a reference copy of the document with all sections, see nature.com/documents/nr-reporting-summary-flat.pdf

Life sciences study design

All studies must disclose on these points even when the disclosure is negative.

Sample size	No statistical method was performed to predetermine sample sizes. For the high-throughput DNA Curtains experiments, the sample size was determined by the molecules acquired and found to adequately sample the behavior of molecules. Our results won't change if increase the sample size indicated our sample size was sufficient. All experiments except for the high-throughput DNA Curtains experiments were repeated at least three times. Figures show one representative experiment.
Data exclusions	No data were excluded in this study.
Replication	This was an in vitro biophysical study, and all replication attempts were successful. All biochemical experiments including SDS-PAGE, in vitro droplet assays, EMSAs, and the biochemistry assay for T7 RNAP, were repeated three times. The high-throughput DNA Curtains experiments were repeated three times.
Randomization	Not relevant, because our work was an in vitro biophysical study.
Blinding	Not relevant, because our work was an in vitro biophysical study.

Reporting for specific materials, systems and methods

We require information from authors about some types of materials, experimental systems and methods used in many studies. Here, indicate whether each material, system or method listed is relevant to your study. If you are not sure if a list item applies to your research, read the appropriate section before selecting a response.

Materials & experimental systems

n/a	Involved in the study	
☐	☒	Antibodies
☐	☒	Eukaryotic cell lines
☒	☐	Palaeontology and archaeology
☒	☐	Animals and other organisms
☒	☐	Human research participants
☒	☐	Clinical data
☒	☐	Dual use research of concern

Methods

n/a	Involved in the study	
☒	☐	ChIP-seq
☒	☐	Flow cytometry
☒	☐	MRI-based neuroimaging

Antibodies

Antibodies used	Monoclonal ANTI-FLAG BioM2 antibody (Sigma F9291-.2mg) was used to prepare anti-FLAG tagged quantum dots.
Validation	The antibody used here was conjugated to DIBO-modified Qdot705 in order to label the proteins with FLAG-tag. 0.125mg antibody/assay was used in the antibody labeling kit (Life technologies, Cat. MP10469).

Eukaryotic cell lines

Policy information about cell lines

Cell line source(s)	HEK 293T human embryonic kidney cells were purchased from ATCC.
Authentication	The cell lines used have been authenticated.
Mycoplasma contamination	Cell lines were tested negative for mycoplasma.
Commonly misidentified lines (See ICLAC register)	No known misidentified lines in ICLAC version 10 database.
