# Supplementary material for: Loci-specific phase separation of FET fusion oncoproteins promotes gene transcription
Source: Nat Commun. 2021 Mar 5;12:1491. doi: 10.1038/s41467-021-21690-7 (PMC7935978; doi:10.1038/s41467-021-21690-7)
Supplement: Supplementary file 12 — Source Data [file 41467_2021_21690_MOESM12_ESM.zip › Source_Data/SourceDataChecklist_ZQ_20210203.pdf]

# Source Data Checklist

## 1. Fig. 1

(1.1) ImageJ (Version: 2.0.0-rc-59/1.5k, Open source image processing software, http://imagej.net/Contributors) was used to analyze all micrographs (Fig. 1a, b, c, d, e, g, h, I, j, and k).

(1.2) All excel documents in the folder "Fig_1" were the FRAP data containing Fig. 1f and 1l. MATLAB software (https://www.mathworks.com/products/matlab.html) was used to load the data and plot Fig. 1f and 1k. MATLAB Software was R2016b (9.1.0.441655), 64-bit (win64), September 7, 2016, License Number: 40504596 (Academic license). This software was installed on the Window operating systems (Windows 10 version 18362.836).

## 2. Fig. 2

(2.1) All experimental data of DNA Curtains were acquired with a custom-built prism-type total internal reflection fluorescence microscope (TIRFM) (Nikon, Inverted Microscope Eclipse Ti-E). The software was CellVision Coolight Technology Version 1.4.0 (Home-made software).

(2.2) ImageJ (Version: 2.0.0-rc-59/1.5k, Open source image processing software, http://imagej.net/Contributors) was used to load and analysis all experimental data of DNA Curtains (Fig. 2c-k).

(2.3) The excel document in the folder "Fig_2" was the data of puncta intensities at the loci (Fig. 2l). The function of "boxplot" in MATLAB software (https://www.mathworks.com/products/matlab.html) was used to plot the boxplot.

## 3. Fig. 3

(3.1) ImageJ (Version: 2.0.0-rc-59/1.5k, Open source image processing software, http://imagej.net/Contributors) was used to load and analysis all experimental data of DNA Curtains (Fig. 3b(i), b(iii), c, d, f, g, h(i), h(ii), and h(iii)).

(3.2) The excel document "Data_Fig_3bii_hv.xlsx" in the folder "Fig_3" was the puncta position distributions in Fig. 3b(ii) and h(v). MATLAB software (https://www.mathworks.com/products/matlab.html) function 'bootci' (bootstrap confidence interval) was used to calculate the error bars for binding position distributions (see Methods).

(3.3) The excel document "Data_Fig_3biv.xlsx" in the folder "Fig_3" was the statistic results in Fig. 3b(iv).

(3.4) The excel document "FRAPdata_Fig3e_PolIICTD_FUSGal4.xlsx" in the folder "Fig_3" were FRAP data of Fig. 3e. MATLAB software was used to load the data and plot this figure.

## 4. Fig. 4

(4.1) ImageJ (Version: 2.0.0-rc-59/1.5k, Open source image processing software, http://imagej.net/Contributors) was used to load and analysis all experimental data of DNA Curtains (Fig. 4a(ii), a(iii), b, c(ii), c(iii), d(ii), d(iii), e(ii), e(iii), g(ii), and g(iii)).

(4.2) The excel document "Data_Fig4f.xlsx" in the folder "Fig_4" was the statistic results in Fig. 4f.

## 5. Fig. 5

For luciferase assays (Methods): the raw data were loaded in the folder of "Fig_5". MATLAB software (https://www.mathworks.com/products/matlab. html) was used to load the data and plot Fig. 5a-d. MATLAB Software was R2016b (9.1.0.441655), 64-bit (win64), September 7, 2016, License Number: 40504596 (Academic license). This software was installed on the Window operating systems (Windows 10 version 18362.836).

## 6. Fig. 6

(6.1) ImageJ (Version: 2.0.0-rc-59/1.5k, Open source image processing software, http://imagej.net/Contributors) was used to load and analysis all experimental data of DNA Curtains (Fig. 6b(i)-(v), c(i), d(i), e(i), f(i), g(i), and h(i)).

(6.2) In the folder "Fig_6", we wrote a MATLAB code "Linyu_Seqcheck_2nd.m" to analyze the consecutive motif / total motif distributions (Fig. 6b(vi)-(vii), c(ii), d(ii), e(ii), f(ii), g(ii) and h(ii)). Instruction for use:

```
%% input
path1 = '/Users/ZhiQi/Nutstore/ZhiQiLab_NutStore/No_01_Projects_NutStore/Project_24_01_LiPL_FUS/No_01_Maintext/NatComm/Revision/Figures/Figure_6/';
Lambdafile = 'lambda25xGGAA.txt';
% Lambdafile = 'lambda13xGGAA.txt';
% Lambdafile = 'lambda9xGGAA.txt';
% Lambdafile = 'lambda7xGGAA.txt';
% Lambdafile = 'lambda5xGGAA.txt';
% Lambdafile = 'lambda3xGGAA.txt';
% Lambdafile = 'lambdaDNA.txt';
```

(6.2.1) Use MATLAB to open "Linyu_Seqcheck_2nd.m", and change "path1" to setup the folder path on your computer.

(6.2.2) Choose the sequence file of DNA substrate. i.e. "lambda25xGGAA.txt' is Lambda DNA containing 25× GGAA. We also prepared all other DNA substrate sequences.

(6.2.3) Setup "bp_distance", which is the linker length between two GGAAs. In the paper, we chose "bp_distance=1" for consecutive motif and "bp_distance=20" for total motif.

(6.2.4) The output is a figure, like Fig. 6b(vi)-(vii), c(ii), d(ii), e(ii), f(ii), g(ii) and h(ii).

(6.2.5) Expected run time for demo on a "normal" desktop computer.

## 7. Supplementary Fig. 1

The folder “Supplementary_Fig_1” contained all uncropped versions of SDS-PAGE images in Supplementary Fig. 1. ImageJ (Version: 2.0.0-rc-59/1.5k, Open source image processing software, http://imagej.net/Contributors) was used to analyze all SDS-PAGE images (Supplementary Fig. 1).

## 8. Supplementary Fig. 3

(8.1) ImageJ (Version: 2.0.0-rc-59/1.5k, Open source image processing software, http://imagej.net/Contributors) was used to load and analysis all experimental data of DNA Curtains (Supplementary Fig. 3a-d).

(8.2) The excel document “Supplementary_Fig_3e.xlsx” in the folder “Supplementary_Fig_3” was the data of the green channel intensity before and after YOYO-1 injection in Supplementary Fig. 3e.

## 9. Supplementary Fig. 4

ImageJ (Version: 2.0.0-rc-59/1.5k, Open source image processing software, http://imagej.net/Contributors) was used to load and analysis all experimental data of DNA Curtains (Supplementary Fig. 4).

## 10. Supplementary Fig. 5

(10.1) ImageJ (Version: 2.0.0-rc-59/1.5k, Open source image processing software, http://imagej.net/Contributors) was used to load and analysis all experimental data of DNA Curtains (Supplementary Fig. 5a).

(10.2) In the folder “Supplementary_Fig_5”, we wrote a MATLAB code “Kym_end_track.m” to track the end of DNA substrates (Supplementary Fig. 5a & Methods). Instruction for use:

(10.2.1) Create several folders: “Kym”, “TethersDatatrack_excel”, “TethersDatatrack_excel_use”, “TethersDatatrack_fig”, “TethersDatatrack_fig_use”, “TethersDatatrack_Matlabfig”.

(10.2.2) The kymograph (like Supplementary Fig. 5a) was loaded into the folder of “Kym”. Here for Demo, we added “No_001.tif” into “Kym”.

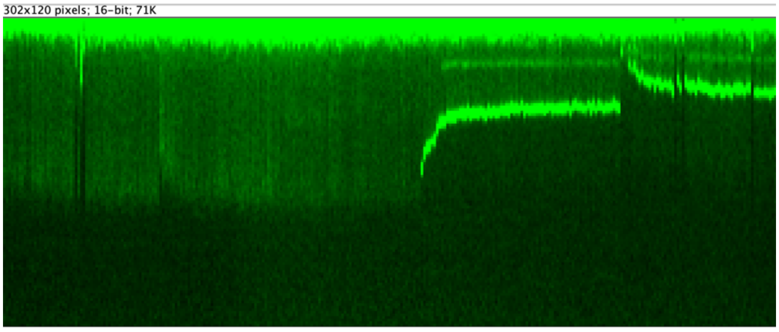


(10.2.3) Use MATLAB to open “Linyu_Seqcheck_2nd.m”, and change “path0” to setup the folder path on your computer. “Kym_length = [1, 302]”, here 302 (pixels) is the length of Kymograph above.

```
%% Input
path0 = '/Users/ZhiQi/Nutstore/ZhiQiLab_NutStore/No_01_Projects_NutStore/Project_24_01_LiPL_FUS/No_01_Maintext/NatComm/Data/Extended_Data_Fig_3/';



Kym_length = [1, 302]; % Linyu, 311
shuttertime  = 5; % second, 5
Method_Num = 3;  % 0, first; then methods >0; or -1

Tether_fig_filename0 = 'No_001';
Tether_fig_filename = [Tether_fig_filename0, '.tif'];

%%%%%%%%%%%
y = 85; % 50 , double length of ini L
T = 24; % frame number after turn on/off? keep the same length, <= 60s, 24
F = 30; % shutter 2s?


L_EMCCD = 16/60; % um, 16/60

LambdaDNA = 48502; % bp
```

(10.2.4) First to run this code by using “Method_Num = 0”. This procedure will run six different tracking methods, and the output is in the folder “TethersDatatrack_fig”. Once we the best tracking condition, run this code again by using “Method_Num = 3” (Here assume the method 3 is the best tracking method). All output data were saved in the folder of “TethersDatatrack_fig_use” (the output figure), “TethersDatatrack_excel_use” (the red color tracking data was saved as an excel file), and “TethersDatatrack_Matlabfig” (the output MATLAB figure).

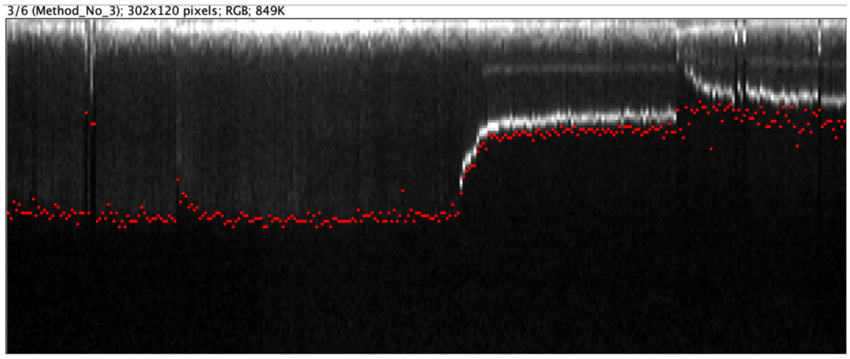


(10.3) The excel documents “Compaction_EWSFLI1.xlsx” and “Compaction_FUSGal4.xlsx” in the folder “Supplementary_Fig_5” were all results of Supplementary Fig. 5b-d. MATLAB software was used to load the data and plot these figures.

## 11. Supplementary Fig. 6

(11.1) ImageJ (Version: 2.0.0-rc-59/1.5k, Open source image processing software, http://imagej.net/Contributors) was used to load and analysis all experimental data of DNA Curtains (Supplementary Fig. 6a-c)

(11.2) The excel document “Data_Supplementary_Fig_6d.xlsx” in the folder “Supplementary_Fig_6” was the statistic results in Supplementary Fig. 6d.

**12. Supplementary Fig. 7**

(12.1) ImageJ (Version: 2.0.0-rc-59/1.5k, Open source image processing software, http://imagej.net/Contributors) was used to load and analysis all experimental data of DNA Curtains (Supplementary Fig. 7a, c, d, and f)

(12.2) The excel document “Data_Supplementary_Fig_7g.xlsx” in the folder “Supplementary_Fig_7” was the statistic results in Supplementary Fig. 7g.

**13. Supplementary Fig. 8**

For ChIP-seq analysis (Methods): Bowtie2 (version 2.3.4) was used for aligning ChIP DNA fragments to human reference genome (hg19). MACS2 (version 3.4) was used for ChIP-seq peak calling. DESeq2 was used for differential gene expression analysis of RNA-seq data. Data: RNA-seq (NCBI SRA059239) and ChIP-seq (NCBI Omnibus GSE99959). The raw data was in in the folder “Supplementary_Fig_8”. For permutation tests of significant overlap between microsatellites and TF, the R library regioneR (1.20.0) was used. All correlations were calculated using Spearman’s rank correlation. LOESS regression (Local Polynomial regression fitting) line was drawn using R library ggplot2.

**14. Supplementary Fig. 9**

The excel document “Supplementary_Fig_9.xlsx” in the folder “Supplementary_Fig_9” was the puncta position distributions in Supplementary Fig. 9. MATLAB function ‘bootci’ (bootstrap confidence interval) was used to calculate the error bars for binding position distributions (Methods).

## 15. Supplementary Fig. 10

(15.1) ImageJ (Version: 2.0.0-rc-59/1.5k, Open source image processing software, http://imagej.net/Contributors) was used to analyze all micrographs (Supplementary Fig. 10a-c)

(15.2) The excel document “Dropsizes_Supplementary_Fig_10d.xlsx” in the folder “Supplementary_Fig_10” was the data of droplet size analysis in Supplementary Fig. 10d.

(15.3) The excel document “Phasediagram_Supplementary_Fig_10e_f_g.xlsx” in the folder “Supplementary_Fig_10” was the data of phase diagrams in Supplementary Fig. 10e-g.
