# Supplementary figures and images for: Loci-specific phase separation of FET fusion oncoproteins promotes gene transcription
Source: Nat Commun. 2021 Mar 5;12:1491. doi: 10.1038/s41467-021-21690-7 (PMC7935978; doi:10.1038/s41467-021-21690-7)

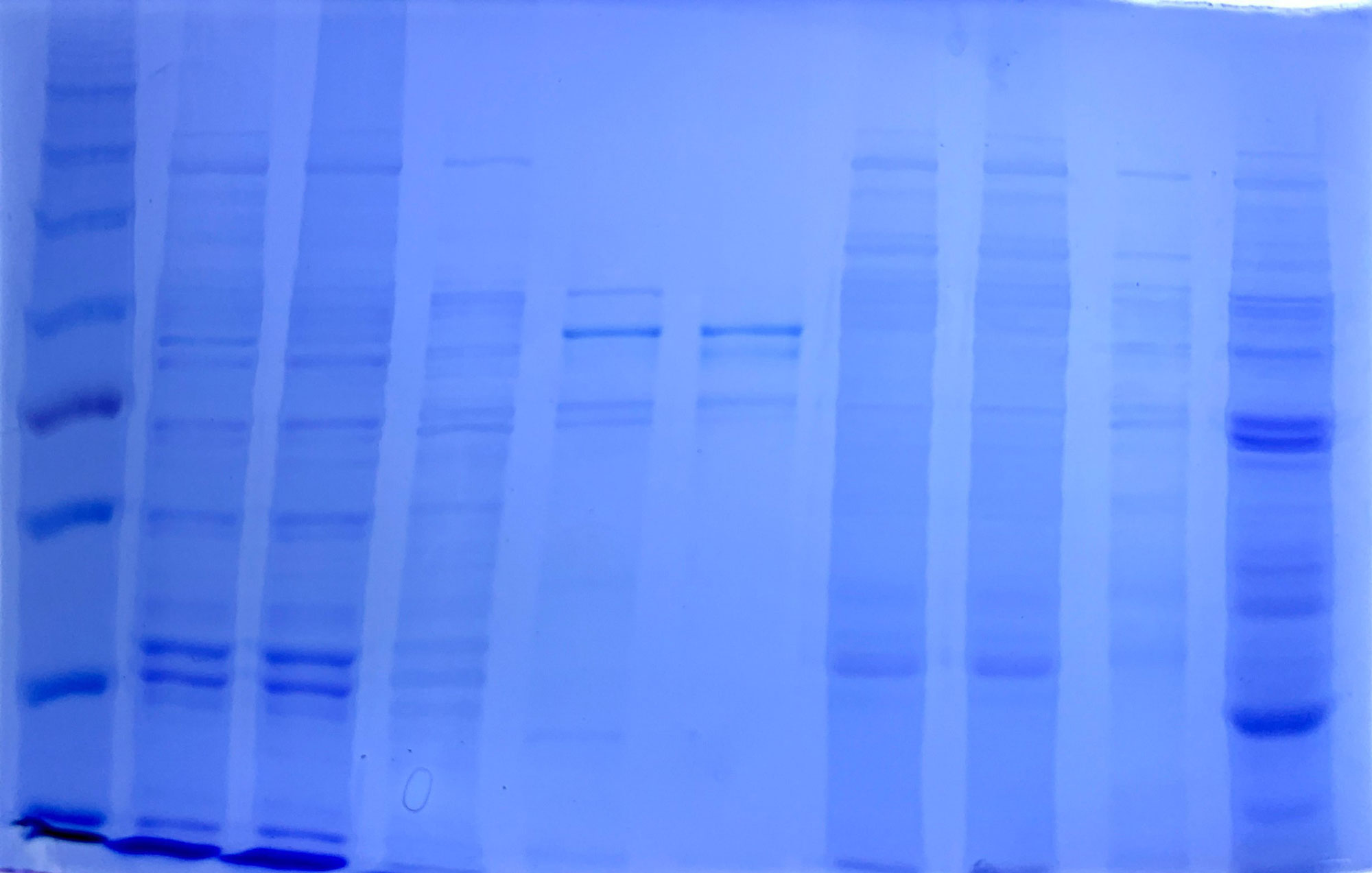

Supplement: Supplementary file 12 — Source Data [file 41467_2021_21690_MOESM12_ESM.zip › Source_Data/Supplementary_Fig_1/SDSPAGE_Supplementary_Fig_1bi_SNAPEWSFLI1.jpg]

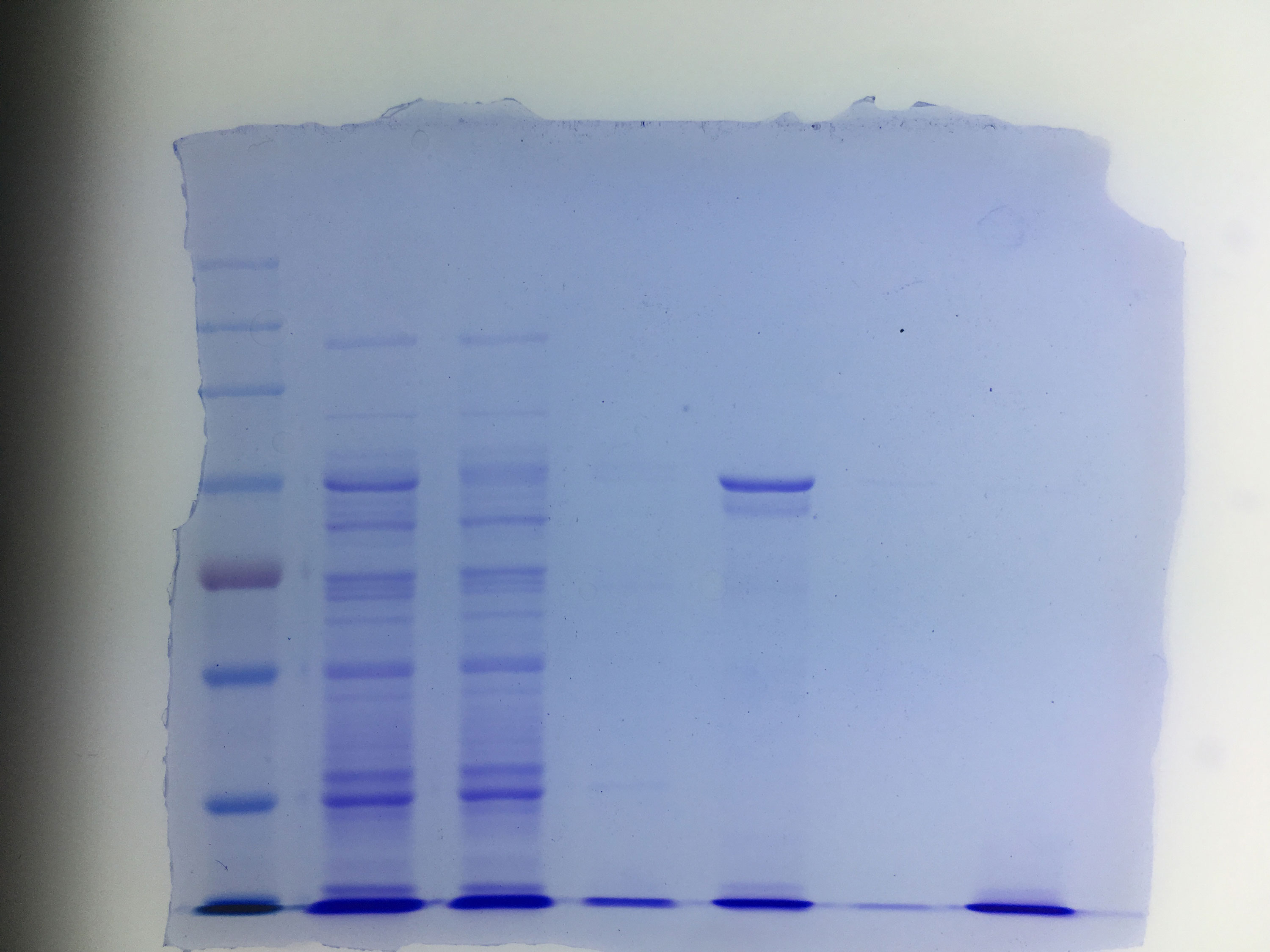

Supplement: Supplementary file 12 — Source Data [file 41467_2021_21690_MOESM12_ESM.zip › Source_Data/Supplementary_Fig_1/SDSPAGE_Supplementary_Fig_1biii_GFPEWSFLI1.jpg]

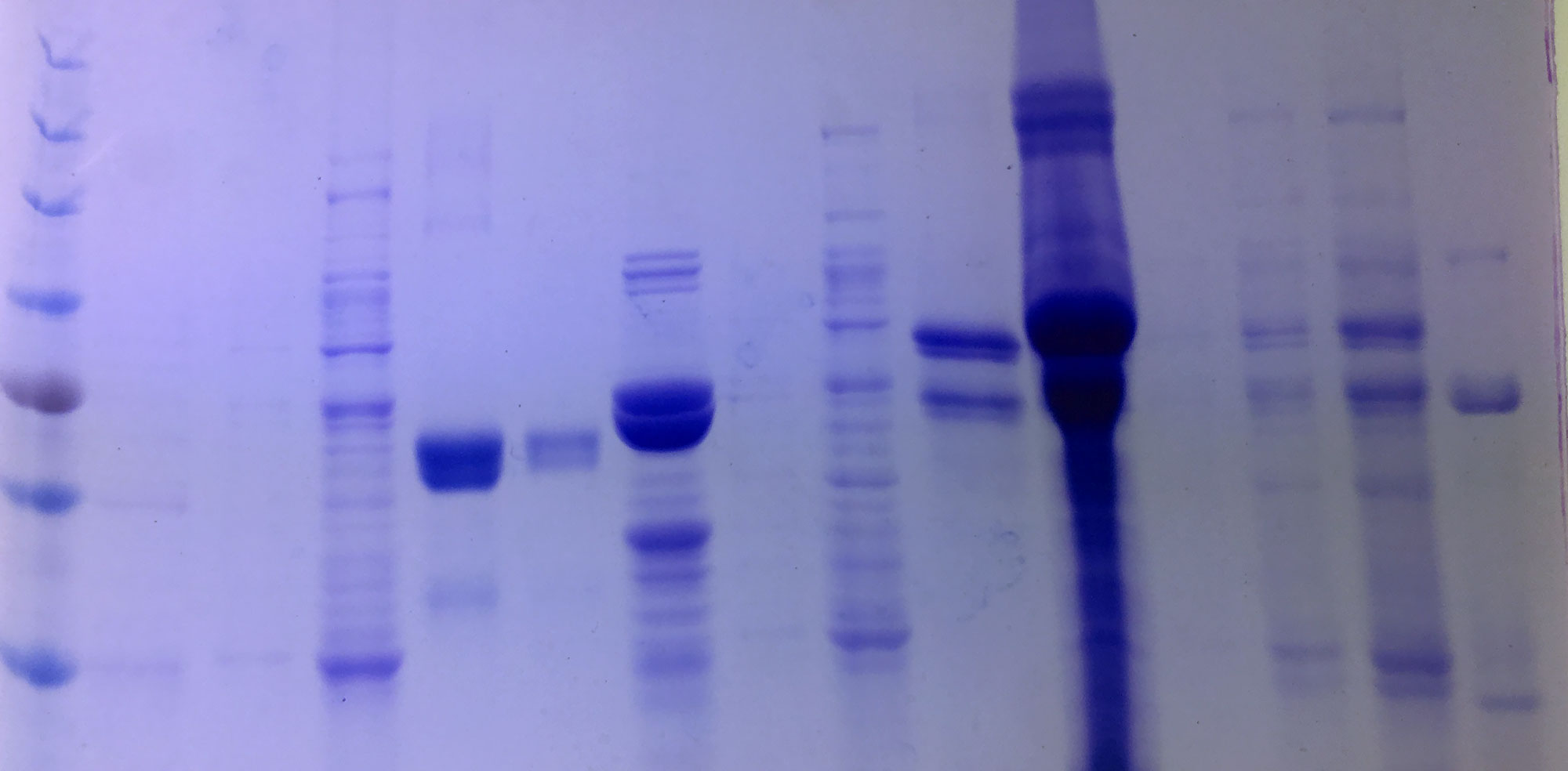

Supplement: Supplementary file 12 — Source Data [file 41467_2021_21690_MOESM12_ESM.zip › Source_Data/Supplementary_Fig_1/SDSPAGE_Supplementary_Fig_1bii_mCherryEWSFLI1.jpg]

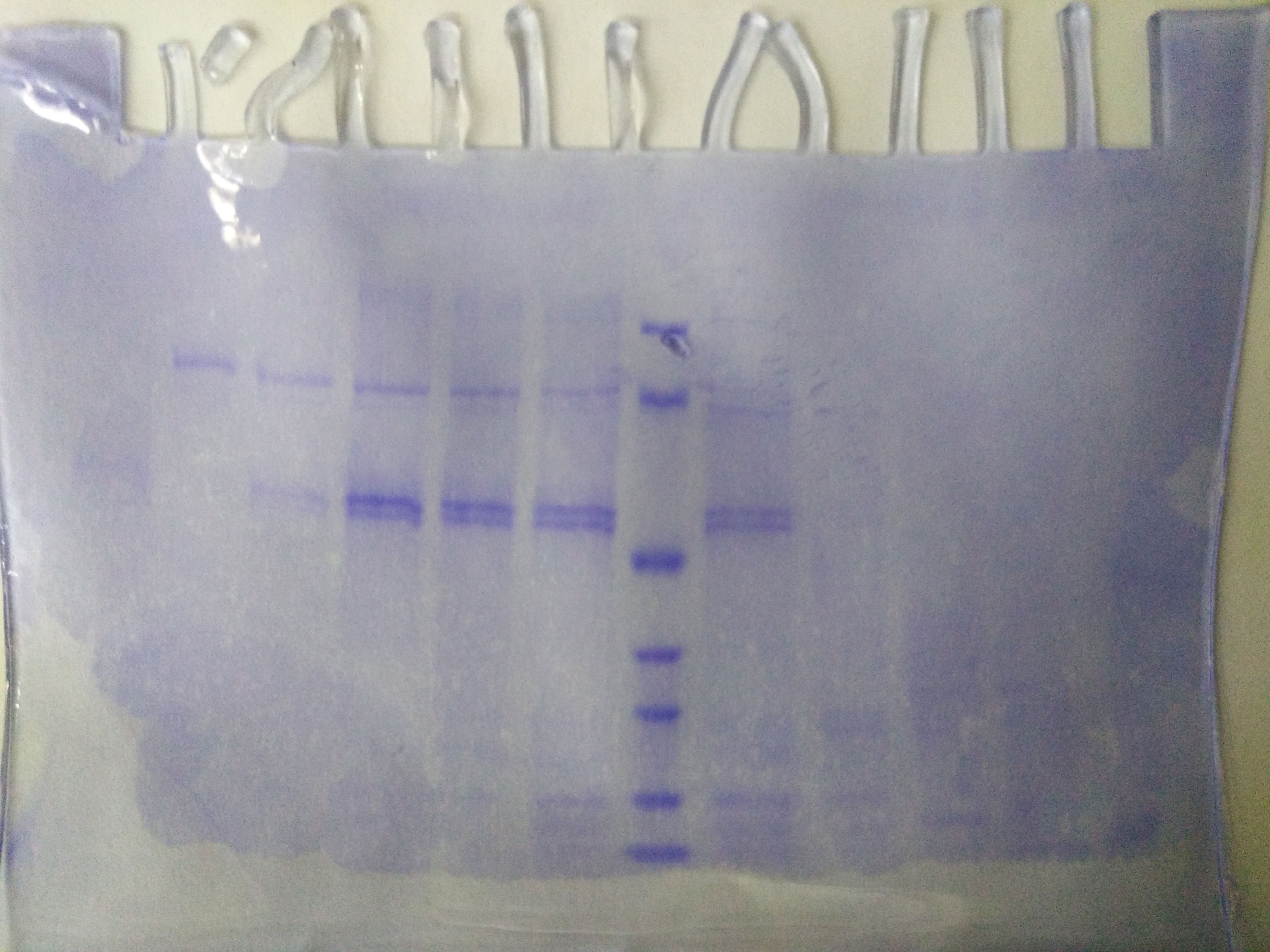

Supplement: Supplementary file 12 — Source Data [file 41467_2021_21690_MOESM12_ESM.zip › Source_Data/Supplementary_Fig_1/SDSPAGE_Supplementary_Fig_1aii_FUSGal4.jpg]

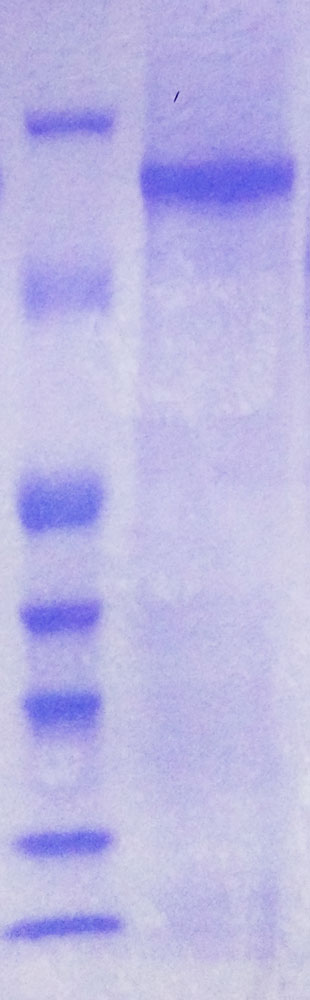

Supplement: Supplementary file 12 — Source Data [file 41467_2021_21690_MOESM12_ESM.zip › Source_Data/Supplementary_Fig_1/SDSPAGE_Supplementary_Fig_1ai_GFPFUSGal4.jpg]

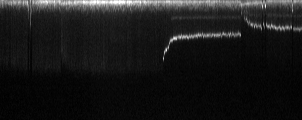

Supplement: Supplementary file 12 — Source Data [file 41467_2021_21690_MOESM12_ESM.zip › Source_Data/Supplementary_Fig_5/Kym/No_001.tif]

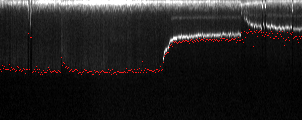

Supplement: Supplementary file 12 — Source Data [file 41467_2021_21690_MOESM12_ESM.zip › Source_Data/Supplementary_Fig_5/TethersDatatrack_fig_use/No_001_3.png]

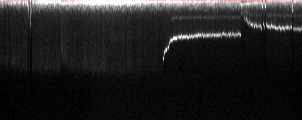

Supplement: Supplementary file 12 — Source Data [file 41467_2021_21690_MOESM12_ESM.zip › Source_Data/Supplementary_Fig_5/TethersDatatrack_fig/Method_No_1.png]

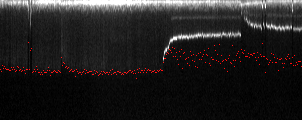

Supplement: Supplementary file 12 — Source Data [file 41467_2021_21690_MOESM12_ESM.zip › Source_Data/Supplementary_Fig_5/TethersDatatrack_fig/Method_No_6.png]

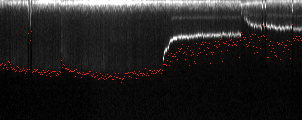

Supplement: Supplementary file 12 — Source Data [file 41467_2021_21690_MOESM12_ESM.zip › Source_Data/Supplementary_Fig_5/TethersDatatrack_fig/Method_No_4.png]
